# ChREBP Is Dispensable for Myofiber Type Switch but Promotes Skeletal Muscle Regeneration

**DOI:** 10.3390/nu18122012

**Published:** 2026-06-21

**Authors:** Junyu Lu, Jian Chen, Guanyu Zhang, Haoxin Ma, Pingxin Sun, Chao Wang, Xinlu Yu, Ke Feng, Chunyan Wang, Chenyi Hu, Xuewei Chen, Wenlin Li

**Affiliations:** 1 Department of Cell Biology, Naval Medical University, Shanghai 200433, China; vaa1234@163.com (H.M.); 13301678592@163.com (P.S.); wangchaosmmu@126.com (C.W.); yuxinludy@163.com (X.Y.); kefeng_bio@foxmail.com (K.F.); chywangnj@163.com (C.W.); 2 Military Medical Sciences Academy, Tianjin 300050, China; cj1999728@163.com (J.C.); zhangguanyu555@foxmail.com (G.Z.); hcy199701@163.com (C.H.); chenxuewei11@sina.com (X.C.)

**Keywords:** skeletal muscle, ChREBP, hypoxia, high-fructose diet, muscle remodeling, muscle regeneration

## Abstract

**Background/Objectives**: The transcription factor carbohydrate response element-binding protein (ChREBP) is a key glucose-sensing regulator that governs glucose and lipid metabolic homeostasis. However, its specific functions in skeletal muscle remain insufficiently clarified. The present study aimed to investigate the roles of ChREBP in skeletal muscle exercise capacity, energy metabolism, and adaptive remodeling, as well as muscle regeneration. **Methods**: We generated a skeletal muscle-specific ChREBP knockout mouse model, and assessed their exercise performance, energy metabolism, skeletal muscle fiber composition, and injury repair capacity. Additionally, hypoxia and high-fructose diet models were established to analyze the function of ChREBP in skeletal muscle adaptive remodeling. C2C12 myoblasts and primary muscle satellite cells were used to explore its effects on myogenic differentiation. **Results**: Genetic deletion of ChREBP induced no detectable alterations in myofiber composition, overall metabolic status, or muscle adaptive remodeling triggered by hypoxia and high-fructose diet. In vitro assays demonstrated that ChREBP overexpression facilitates C2C12 myogenic differentiation. Adeno-associated virus-mediated ChREBP overexpression enhanced histological markers of regeneration, including desmin-positive regenerative area and the cross-sectional area of newly formed myofibers after cardiotoxin-induced injury. **Conclusions**: Collectively, our experimental data indicate that ChREBP is largely dispensable for maintaining basal skeletal muscle homeostasis and stress-induced adaptive remodeling. Meanwhile, this study identifies a previously unrecognized regulatory role of ChREBP in the processes of skeletal muscle damage repair and post-injury regeneration.

## 1. Introduction

Skeletal muscle functions not only as a locomotor apparatus but also as a vital metabolic and endocrine organ that significantly influences whole-body energy homeostasis. The composition of myofibers and energy metabolism constitute the two primary determinants of skeletal muscle function [1,2,3]. Based on their contractile and metabolic properties, skeletal muscle fibers are classified into slow-twitch (type I) and fast-twitch (type II) myofibers. Type I myofibers are rich in mitochondria and rely largely on mitochondrial oxidative metabolism, whereas type II myofibers generally contain fewer mitochondria and have lower oxidative capacity. Myofiber type switching and metabolic reprogramming constitute the adaptive remodeling of skeletal muscle in response to physiological or pathological stimuli. Disruption of this coordinated regulatory mechanism can lead to physiological dysfunction.

Skeletal muscle exhibits significant plasticity to satisfy its energetic and contractile requirements. Environmental challenges, particularly hypoxia, are well known to alter myofiber composition and metabolic homeostasis. Several studies have investigated the impact of environmental and pathological hypoxic conditions on myofiber type, yet published observations from these studies remain inconsistent [4,5,6].

Similarly, nutritional conditions act as a central factor governing the adaptive shifts in myofiber phenotypes [7,8,9]. In recent decades, the continuous rise in dietary fructose intake has drawn widespread public health attention. Epidemiological investigations have noted that long-term overconsumption of dietary fructose correlates closely with the onset of metabolic syndrome, a disorder marked by a cluster of symptoms including obesity, insulin resistance, hyperglycemia, dyslipidemia, hypertension, non-alcoholic fatty liver disease, gout and hyperuricemia [10,11,12,13,14]. Notably, published data regarding how a high-fructose diet (HFrD) regulates skeletal muscle fiber phenotype remain contradictory. Several studies have demonstrated that hyperactivated sterol regulatory element-binding protein (SREBP) signaling driven by excess fructose favors an oxidative myofiber phenotype [15], whereas other studies in juvenile Iberian pigs show that combined high-fructose and high-fat feeding reduces muscle oxidative capacity [16]. To date, direct evidence linking high-fructose intake to myofiber-type remodeling is still limited.

ChREBP, also known as MLX-interacting protein-like (MLXIPL), serves as an essential transcription factor in the regulation of carbohydrate metabolism and lipogenesis [17,18,19,20,21,22,23,24]. Although ChREBP is well-established as an indispensable regulator of glycolipid homeostasis in the liver and adipose tissue, its regulatory role in highly metabolic skeletal muscle remains poorly understood. Previous studies have shown that global ChREBP knockout mice exhibit significantly reduced muscle mass [25]. An in vitro study has suggested a role for ChREBP in muscle fiber type plasticity, showing that it partially mediates the impact of high glucose on the metabolic transition from slow-twitch to fast-twitch fibers [26]. However, these findings have not been further validated using muscle-specific knockout models.

Accumulating evidence has confirmed that hepatic and intestinal ChREBP are highly responsive to fructose. Fructose-mediated activation of ChREBP may contribute to hepatic steatosis, hypertriglyceridemia, and both hepatic and peripheral insulin resistance [27,28,29]. Consistently, ChREBP-deficient mice develop fructose intolerance and rapidly deteriorate within one to two weeks when maintained on a HFrD [30,31]. Separately, prior work has illustrated that ChREBP participates in reshaping metabolic profiles within tumor cells exposed to hypoxic microenvironments. Silencing ChREBP in colon cancer cells suppresses aerobic glycolysis and inhibits cell proliferation and metastasis [32]. Given that hypoxic exposure and excessive fructose consumption represent two representative metabolic stressors capable of stimulating ChREBP signaling and disturbing systemic metabolic homeostasis, these two intervention models provide a valuable platform to elucidate the biological roles and downstream pathways of ChREBP in skeletal muscle.

Beyond adaptive metabolic remodeling, skeletal muscle retains potent regenerative capacity to restore tissue architecture following acute injury. This regenerative process is principally orchestrated by resident muscle satellite cells (MuSCs). Upon muscle damage or exposure to extracellular cues, quiescent MuSCs become activated, undergo clonal expansion, and ultimately differentiate to fuse with damaged endogenous myofibers or to form nascent myotubes. Accordingly, MuSCs maintain basal skeletal muscle homeostasis and mediate stress-triggered adaptive remodeling. Post-injury muscle repair is a highly coordinated process involving myofiber degeneration, inflammatory cell infiltration, de novo myogenesis and tissue maturation. MuSCs, immune cells and interstitial stromal cells act synergistically to facilitate tissue repair [33,34]. Because metabolic regulators frequently coordinate MuSCs activation and differentiation, it remains unclear whether ChREBP participates in muscle regeneration independent of metabolic adaptation.

Collectively, we hypothesize that ChREBP modulates multiple physiological processes in skeletal muscle, including exercise performance, adaptive remodeling and post-injury regeneration. To test this hypothesis, we generated muscle-specific ChREBP knockout (ChMKO) mice to dissect the basal physiological functions of muscular ChREBP in skeletal muscle. We then induced metabolic stress via hypoxic exposure and high-fructose feeding, and analyzed changes in myofiber composition to determine whether ChREBP regulates stress-triggered muscle remodeling. We further used C2C12 myoblasts and an adeno-associated virus (AAV)-mediated local ChREBP overexpression mouse model to characterize ChREBP’s role in skeletal muscle regeneration following acute injury.

## 2. Materials and Methods

### 2.1. Animals

C57BL/6J wild-type mice were purchased from Beijing Vital River Laboratory Animal Technology Co., Ltd. All mice underwent a one-week acclimation period before experimental manipulation. Mice were housed in a specific pathogen-free barrier facility at a controlled temperature of 23–25 °C with a 12 h light/12 h dark cycle. All animal experiments strictly followed the NIH Guidelines for the Care and Use of Laboratory Animals and were approved by the Institutional Animal Care and Use Committee of the Military Medical Sciences Academy, Tianjin, China (IACUC No. AMMS-04-2022-201).

Two distinct experimental cohorts were established using C57BL/6J wild-type mice. The first set involved three independent experimental models: hypoxia exposure, short-term HFrD (2 weeks), and long-term HFrD (3 months). Each model contained paired intervention and control groups (*n* = 10 per group). The second set divided wild-type mice into four groups according to AAV-ChREBP injection and Cardiotoxin (CTX) administration (*n* = 5 per group).

**Figure 1:**
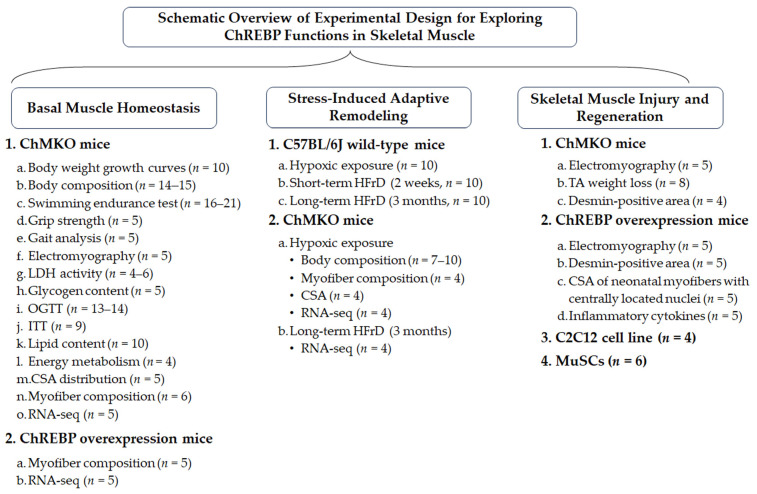
Experimental workflow and sample size summary. This schematic illustrates the three major research components of the present study: basal muscle homeostasis, stress-induced adaptive remodeling, and skeletal muscle injury and regeneration. It summarizes the animal and cell models, experimental interventions and functional assays, with the number of biological replicates (*n*) indicated for each measurement. LDH, lactate dehydrogenase; OGTT, oral glucose tolerance test; ITT, insulin tolerance test; CSA, cross-sectional area. TA, tibialis anterior.

ChMKO mice were bred in-house, with littermate ChREBP-floxed control mice (hereafter referred to as F/F) serving as controls. Mice of each genotype were randomly assigned to the hypoxia exposure, long-term HFrD and CTX treatment groups. The sample size of ChMKO mice was adjusted according to breeding availability and experimental requirements (Figure 1).

Unless otherwise specified, male mice aged 8–10 weeks were used in all experiments. Tissues were collected upon completion of experimental interventions. Mice were anesthetized via isoflurane inhalation. Skeletal muscle, liver and intestinal tissues were then dissected from standardized anatomical sites. To minimize technical variation, tissues with consistent wet weights were harvested for each downstream assay. For gastrocnemius (GC) muscle biochemical detection, 20–30 mg fresh tissue was collected per mouse after trimming away tendons, fascia and fat. All samples were rinsed with ice-cold phosphate-buffered saline (PBS) to clear blood and tissue debris, gently blotted dry with filter paper, and then either snap-frozen in liquid nitrogen for −80 °C storage or fixed instantly for morphological evaluation. At the end of experiments, mice were euthanized by isoflurane anesthesia followed by cervical dislocation, with no prolonged housing. This unified euthanasia protocol was used throughout the study.

### 2.2. Generation of ChMKO Mice

ChREBP-flox mice were generated by gene targeting as previously described [30]. Briefly, ChREBP-flox mice were engineered with loxP sites flanking exon 8. Cre-mediated recombination results in the deletion of exon 8, which consequently induces a frameshift mutation affecting exons 9 through 17 and leading to premature termination of translation. The resultant loss of the *C*-terminal bHLH/ZIP (basic helix–loop–helix/leucine zipper) domain inhibits ChREBP from binding to the promoter regions of target genes, thereby nullifying its transcriptional regulatory function. Muscle creatine kinase-Cre (MCK-Cre) mice were obtained from Shanghai Southern Model Organisms Co., Ltd. (Shanghai, China). ChMKO mice were generated by crossing ChREBP-flox mice with MCK-Cre mice.

### 2.3. Hypoxic Exposure

Before hypoxic treatment, all mice underwent a one-week acclimation period under normoxic housing conditions to stabilize their basal physiological state. The hypoxic intervention was carried out in two separate experiments, with *n* = 10 for C57BL/6 mice and *n* = 7–10 for ChMKO mice per group. Mice were maintained inside a hypoxic chamber (Shanghai Yuyan Scientific Instrument Co., Ltd., Shanghai, China) for 2 weeks of continuous treatment, simulating an altitude of ~5500 m. The chamber was stably controlled at atmospheric pressure of 46–49 kPa with a partial oxygen pressure of approximately 10 kPa. Upon completion of hypoxia exposure, all mice were immediately transferred back to the standard housing conditions for subsequent observations and sample collection.

### 2.4. High-Fructose Diet

HFrD intervention was performed in two independent experimental stages, including a short-term 2-week regimen and a long-term 3-month regimen. C57BL/6 mice were used in both short-term and long-term interventions, with *n* = 10 per group. For RNA-seq assays after HFrD feeding, *n* = 4 ChMKO mice per group were included. All experimental mice were maintained under consistent housing conditions with ad libitum access to food and water. Mice with identical baseline age and body weight were randomly allocated to either the normal chow diet group or the HFrD group. The customized HFrD consisted of 20% protein, 68% fructose, and 12% fat by calories (Xiaoshuyoutai Biotechnology Company, Beijing, China). Formal quantification and statistical comparison of daily food intake between groups were not prioritized for this study; routine daily animal surveillance revealed no overt disparities in feeding behavior among experimental groups.

### 2.5. Swimming Endurance Test

Mice were placed in transparent plastic buckets filled with water maintained at 24–26 °C with a depth of 25–30 cm. Mice were grouped by genotype: F/F (*n* = 16) and ChMKO (*n* = 21). Before the formal swimming test, mice were gradually acclimated to swimming over three consecutive days to reduce stress. The daily swimming duration was increased progressively: 5 min on Day 1, 10 min on Day 2, and 15 min on Day 3. Water temperature, depth and ambient conditions remained consistent throughout acclimation and formal trials.

For the formal test, a load equivalent to 3% of each mouse’s body weight was attached to the tail during swimming. Exhaustion was defined as the point when a mouse failed to keep its nose above the water surface for 5 consecutive seconds. Animals were immediately rescued once exhausted.

### 2.6. Grip Strength

Mouse grip strength was measured using a digital grip strength meter (PL-1000, Vokes Biotech Co., Ltd., Shanghai, China). Mice were divided by genotype, with *n* = 5 per group. Briefly, mice were placed to grasp the grid with their forepaws in a horizontal position. The tail was pulled backward smoothly until the grip was released, and the grip strength was recorded. Three consecutive trials were conducted per mouse; the mean value was calculated and normalized to body weight.

### 2.7. Gait Analysis

Gait parameters were assessed using a video-based gait analysis and processing system (Zhongshidichuang Science and Technology Development Co., Ltd., Beijing, China). Mice were divided by genotype, with *n* = 5 per group. Briefly, mice were placed at the entrance of a treadmill and allowed to traverse the runway autonomously and continuously. A high-frequency camera positioned above the runway simultaneously recorded footprint information, gait videos, and plantar pressure data. The system’s companion software automatically analyzed video and pressure data to identify individual paw print contours and calculate single-paw parameters and coordination indices. Valid gait sequence data were averaged for subsequent statistical analysis.

### 2.8. Electromyography (EMG)

EMG testing was performed using a biological signal acquisition system (MD3000, Zhenghua Biological Instrument Equipment Co., Ltd., Hefei, China). Mice were grouped by genotype, with *n* = 5 per group. Briefly, following anesthesia with isoflurane, the skin over the hindlimb was incised to expose the TA muscle. A 4-0 suture was passed through the TA muscle and secured (for signal detection). Fine needles were inserted into the proximal and distal ends of the TA muscle and connected to alligator clips (for voltage output). The stimulation voltage was adjusted to determine the optimal voltage for eliciting maximal muscle tension. Contraction force recovery (%) was calculated as the ratio of the electromyographic signal amplitude of the CTX-injured leg to that of the contralateral control leg of the same mouse.

### 2.9. Muscle Injury and Regeneration Model

To establish an acute muscle injury model, mice were anesthetized with isoflurane, and 50 μL of 20μM CTX (#217503, Sigma, St. Louis, MO, USA) was injected into the TA of one leg, with the contralateral TA receiving an equal volume of PBS as a control. Each group included *n* = 5 mice. TA muscle weight loss was normalized to the contralateral PBS-injected TA muscle.

### 2.10. AAV Production and Intramuscular Injection

AAV9-ChREBP and AAV9-green fluorescent protein (GFP) control viruses were constructed and packaged by Shanghai OBiO Technology Corp., Ltd. (Shanghai, China). Viruses used the Cytomegalovirus (CMV) promoter to drive full-length mouse ChREBP expression. To achieve local overexpression in vivo, 8–10-week-old male mice received a single intramuscular AAV injection into the designated muscle at a dose of 5 × 10^10^ vg per muscle. Each group included n = 5 mice. Mice were euthanized and tissues were harvested for subsequent analyses at 14 days post-injection (dpi).

### 2.11. Body Composition and Metabolic Assessments

Body composition of mice was quantified using an InAlyzer dual-energy X-ray absorptiometry analyzer (Medikors, Seongnam, Republic of Korea), a fully validated instrument dedicated to body composition measurement in small laboratory rodents. Plasma cholesterol, plasma and GC muscle triglyceride (TG) were measured as previously reported [35]. Specifically, TG was measured using a colorimetric kit (Cat. No. TR0100, Sigma, St. Louis, MO, USA), and total cholesterol (TC) was determined with a fluorometric Amplex™ Red Cholesterol Assay Kit (Cat. No. A12216, Invitrogen, Waltham, MA, USA). GC Muscle glycogen was detected with a glycogen assay kit (D799397-0050, Sangon Biotech, Shanghai, China). Plasma LDH activity was measured by a L-Lactate Dehydrogenase Assay Kit (P0393S, Beyotime, Shanghai, China). Grouping by genotype: F/F and ChMKO. TC and TG, *n* = 10 per group; GC muscle glycogen, *n* = 5 per group; LDH activity, *n* = 4–6 per group.

OGTT and ITT were performed after a 6 h fast. Mice were grouped by genotype, with *n* = 13–14 per group for OGTT and *n* = 9 per group for ITT. Glucose was administered by gavage (2.0 g/kg body weight) for OGTT, or insulin was administered via i.p. injection (1.0 U/kg body weight) for ITT. Blood glucose was measured at 0, 15, 30, 60, and 120 min after glucose or insulin administration, using a glucose monitor (One Touch Ultra, Lifescan, Johnson & Johnson, Milpitas, CA, USA).

### 2.12. Cell Culture, Differentiation, and Adenoviral Transduction

C2C12 myoblasts were purchased from ATCC (Manassas, VA, USA) and cultured in growth medium (Dulbecco’s Modified Eagle Medium (DMEM) containing 10% fetal bovine serum (FBS, Gibco, Waltham, MA, USA)). To establish stable cell lines, C2C12 myoblasts were transduced with control and ChREBP overexpressing retroviruses, followed by puromycin selection (2 µg/mL). Myotube differentiation was initiated by switching C2C12 myoblasts at ~80% confluency to differentiation medium (DMEM containing 2% horse serum (Gibco, Waltham, MA, USA)). The differentiation medium was replenished every 2 days until terminal differentiation. Cells were subsequently harvested for immunofluorescence staining and gene expression analysis.

MuSCs were isolated and cultured as previously described [36]. Briefly, one-month-old mice were euthanized under sterile conditions. Hindlimb skeletal muscles, including the GC, TA and quadriceps, were dissected and cleared of tendons, fat and connective tissues. The tissues were minced thoroughly and sequentially digested with collagenase II (#17101015, Gibco, Waltham, MA, USA) and dispase II (D4693, Sigma, St. Louis, MO, USA). Following digestion, the cell suspension was filtered, purified via differential plating, centrifuged and resuspended for primary MuSCs culture and subsequent experiments.

MuSCs were cultured on Geltrex (A1413302, Gibco, Waltham, MA, USA)-coated dishes in cytokine cocktail medium (DMEM containing 10% FBS, 5 ng/mL interleukin-1α (IL-1α, #315-05, Peprotech, Cranbury, NJ, USA), 5 ng/mL interleukin-13 (IL-13, #210-13, Peprotech, Cranbury, NJ, USA), 10 ng/mL interferon-γ (IFN-γ, #315-01A, Peprotech, Cranbury, NJ, USA), 10 ng/mL tumor necrosis factor-α (TNF-α, #211-11A, Peprotech, Cranbury, NJ, USA), and 2.5 ng/mL basic fibroblast growth factor (bFGF, #450-33, Peprotech, Cranbury, NJ, USA)). For serial expansion, 10,000 cells were seeded in a 3.5 cm dish and passaged every 2 days. For myogenic differentiation, MuSCs were seeded at 100,000 cells per well in 6-well plates. At 60–70% confluence, cells were infected with 5 × 10^6^ plaque-forming unit (PFU) adenovirus-Cre (Ad-Cre) for 8 h, then cultured in differentiation medium (2% horse serum in DMEM). Cells were harvested for analysis 72 h post-induction.

### 2.13. RNA Sequencing (RNA-Seq) Analysis

Total RNA was isolated from tissue samples using TRIzol reagent. RNA integrity and purity were assessed with an Agilent 2100 Bioanalyzer (Agilent Technologies, Palo Alto, CA, USA), and only samples with qualified RNA integrity were used for subsequent library construction. Each group included at least 4 independent biological replicates. Complementary DNA library preparation and high-throughput transcriptome sequencing were conducted by Gene Denovo Biotechnology Co., Ltd. (Guangzhou, China) on the Illumina NovaSeq 6000 platform. Raw reads were quality-controlled and trimmed using fastp (v0.18.0) to obtain high-quality clean reads, which were then aligned to the reference genome with HISAT2. Differential expression analysis was carried out via DESeq2. Genes with |log_2_ fold change| ≥ 1 and *p* < 0.05 were defined as significantly differentially expressed genes (DEGs). Given that this analysis was intended for functional screening, multiple-testing correction was not applied. No obvious batch effects were detected among samples, so no additional batch-effect correction was performed. Data visualization and functional enrichment analyses of DEGs were implemented in R software (v4.4.1) with the packages clusterProfiler v4.17.0, enrichplot v1.29.2, topGO, and VennDiagram.

### 2.14. mRNA Expression Analysis

Total RNA was extracted from tissue or cells using the TRIZOL method. The concentration and purity of the extracted RNA were assessed via spectrophotometry (NanoDrop; Thermo Scientific, Waltham, MA, USA). Complementary DNA (cDNA) was synthesized using the First Strand cDNA Synthesis Kit Rever Tra Ace-α (FSK-101, Toyobo, Osaka, Japan). mRNA expression was measured by quantitative real-time polymerase chain reaction (qRT-PCR) using the SYBR Green dye-based assay with the *36b4* gene as internal control in every plate. Each sample was run in technical duplicates. Replicate pairs with a threshold cycle (Ct) difference greater than 0.5 were excluded from subsequent data analysis. The qRT-PCR primer sequences are listed in Table S1.

### 2.15. Immunoblotting Analysis

Protein lysates from skeletal muscle tissues and cultured cells were quantified using a bicinchoninic acid (BCA) protein assay kit (P0010, Beyotime, Shanghai, China). Equal protein samples were separated by sodium dodecyl sulfate-polyacrylamide gel electrophoresis (SDS-PAGE) and electro-transferred to polyvinylidene difluoride (PVDF) membranes (#3010040001, Millipore, Burlington, MA, USA). After blocking, membranes were then incubated with corresponding primary antibodies overnight at 4 °C. Immunoreactive bands were visualized with SuperSignal™ West Pico PLUS Chemiluminescent Substrate (#34580, Invitrogen, Waltham, MA, USA), and images were captured with an Amersham Imager 680 (AI680, Cytiva, Marlborough, MA, USA). The primary antibodies used were as follows: ChREBP (1:1000, NB400-135, Novus, Littleton, CO, USA), α-TUBULIN (1:5000, 66031-1-Ig, PROTEINTECH, Wuhan, China), MYOG (1:1000, ab124800, Abcam, Cambridge, UK), MYOD1(1:1000, 18943-1-AP, PROTEINTECH, Wuhan, China), MyHC (1:1000, ab37484, Abcam, Cambridge, UK), DESMIN (1:1000, #5332, CST, Danvers, MA, USA) and FLAG-tag (1:1000, 6608-4-Ig, PROTEINTECH, Wuhan, China).

### 2.16. Immunofluorescence Staining and CSA Calculation

Muscle tissues were fixed with specialized muscle fixative solution (G1111, Servicebio, Wuhan, China), followed by paraffin embedding and sectioning. Immunohistochemical analysis of myofiber type was performed on muscle sections by overnight incubation at 4 °C with the following primary antibodies: slow skeletal myosin heavy chain (MHC) (GB111857, Servicebio, Wuhan, China), fast skeletal MHC (GB112130, Servicebio, Wuhan, China). Following wheat germ agglutinin (WGA) staining for sarcolemmal labeling of muscle fibers, the CSA of individual myofibers was quantified using Fiji (v2.14.0, based on ImageJ2 v1.54p, National Institutes of Health, USA). Detailed sample sizes for these analyses across different interventions are presented in Figure 1.

### 2.17. ELISA Assays

Muscle tissues were homogenized in ice-cold PBS with protease inhibitors, centrifuged at 5000× *g* for 10 min at 4 °C, and supernatants were collected. Mice were divided into AAV-GFP and AAV-ChREBP groups, with *n* = 5 per group. IL-13 (#ED-20167), TNF-α (#ED-20852), and IFN-γ (#ED-22688) were measured using commercial mouse ELISA kits (Amoylunchangshuo Biotech Co., Ltd., Xiamen, China) according to the manufacturer’s instructions.

### 2.18. Statistics Analysis

All data are presented as mean ± standard deviation (SD). Normality was confirmed by the Shapiro–Wilk test, and homogeneity of variances was evaluated with Levene’s test. Unpaired two-tailed Student’s *t*-test (with Welch’s correction for unequal variances) was used for two-group comparisons, with Cohen’s *d* for effect sizes. For multiple-group comparisons, one-way analysis of variance (ANOVA) was performed, followed by Bonferroni’s test for targeted comparisons versus a control group. For repeated-measures data (e.g., OGTT, ITT), a repeated-measures ANOVA was adopted, with Bonferroni’s test for time point comparisons. For two-factor interactive designs (e.g., genotype × diet, genotype × hypoxia interventions), two-way ANOVA followed by Bonferroni’s test was performed. For myofiber proportional distribution across CSA bins, two-way ANOVA was followed by post hoc two-stage linear step-up Benjamini–Krieger–Yekutieli false discovery rate (FDR) correction (BKY, Q = 0.05). Partial $\eta_{p}^{2}$ and Cohen’s *d* were used to assess overall and pairwise effect sizes across ANOVA analyses. Non-normally distributed datasets were analyzed using non-parametric tests: Kruskal–Wallis H test followed by Dunn’s post hoc test was used for overall multi-group comparisons (e.g., *ChREBP* abundance in different skeletal muscle groups), with effect sizes reported as *ε*^2^ for overall effects and *r* for pairwise comparisons. Post hoc power analysis was additionally conducted at two-sided α = 0.05 to evaluate the detection capability of the adopted sample sizes. A *p* value < 0.05 was considered statistically significant. All statistical analyses were performed using GraphPad Prism 10.0.

The sample size for this study was determined by a combination of a priori power analysis (two-sided α = 0.05, power = 0.8) based on pre-experimental data and previous experimental experience from our research group. Calculation results indicated that 4–6 animals per group were sufficient to detect the anticipated intergroup effect magnitudes. This sample size is in line with typical animal numbers commonly adopted in published studies focusing on murine skeletal muscle phenotypes and molecular markers.

## 3. Results

### 3.1. Generation of the ChMKO Mouse Model

To investigate the role of ChREBP in muscle tissue, we initially assessed its expression across various muscle groups in C57BL/6 mice. ChREBP has two major isoforms, ChREBP-α and ChREBP-β. The mRNA level of *ChREBP-α* was comparable among multiple skeletal muscle types, including GC, TA, extensor digitorum longus (EDL), soleus (SOL), and diaphragm (DIA) muscles. In contrast, *ChREBP-β* was predominantly expressed in adipose tissues and hardly detectable in skeletal muscle (Figure S1).

**Figure 2:**
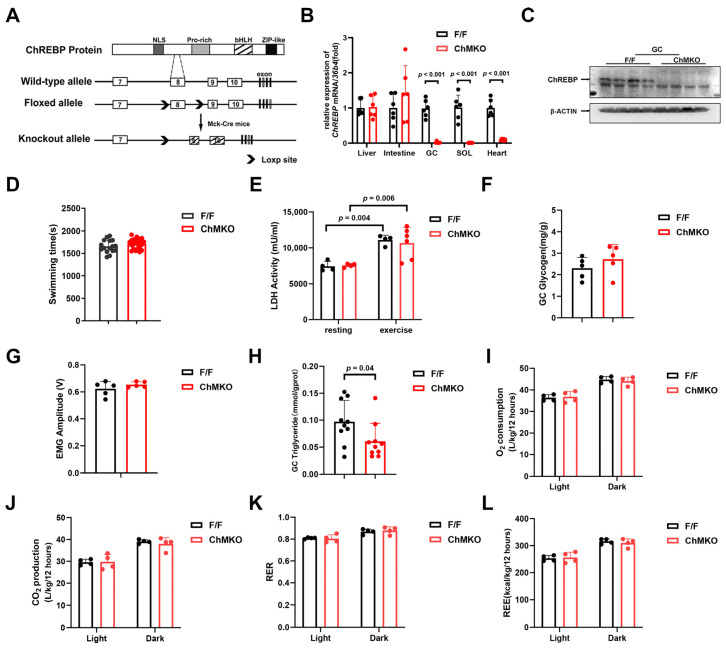
Generation of the ChMKO mouse model. (**A**) Schematic diagram illustrating the construction of ChMKO mice. NLS, nuclear localization signal. (**B**,**C**) Validation of the specificity and efficiency of ChREBP knockout by Western blot analysis and qRT-PCR, *n* = 6 biological replicates (individual mice), 2 technical replicates per sample, Welch’s *t*-test, *d* value for F/F vs. ChMKO: GC (4.22), SOL (3.93), heart (4.29), power = 1. (**D**) Exhaustive swimming test, F/F, *n* = 16; ChMKO, *n* = 21 biological replicates, *d* = 0.44. (**E**) Quantification of plasma LDH activity at baseline rest and immediately post-exhaustive swimming, F/F resting, *n* = 4; F/F exercise, *n* = 4; ChMKO resting, *n* = 4; ChMKO exercise, *n* = 6 biological replicates, 2 technical replicates per sample, two-way ANOVA followed by Bonferroni’s multiple comparisons test, *d* value for F/F vs. ChMKO: resting (0.23), exercise (0.24). (**F**) Glycogen content in the GC muscle of resting ChMKO mice, normalized to muscle wet weight, *n* = 5 biological replicates, 2 technical replicates per sample, *d* = 0.70. (**G**) EMG analysis of the TA muscle in ChMKO mice, *n* = 5 biological replicates, *d* = 0.76. (**H**) TG content in the GC muscle of ChMKO mice, normalized to total protein content, *n* = 10 biological replicates, 2 technical replicates per sample, unpaired two-tailed Student’s *t*-test, *d* = 0.99, power = 0.83. (**I**–**L**) Analysis of O_2_ consumption, CO_2_ production, RER, and REE in ChMKO mice using the TSE LabMaster system. Data were recorded during the light cycle (7:00–19:00) and dark cycle (19:00–7:00), *n* = 4 biological replicates. *d* value for F/F vs. ChMKO: O_2_ consumption (0.20, 0.42), CO_2_ production (0.08, 0.50), RER (0.17, 0.33), REE (0.18, 0.46) at light and dark cycle, respectively.

We generated a skeletal muscle-specific knockout model [30] (Figure 2A). Quantitative analysis of total *ChREBP* mRNA revealed markedly reduced expression within GC, SOL, and cardiac muscle tissues (*p* < 0.001), compared with littermate control mice. In contrast, no obvious changes were detected in the liver and small intestine (Figure 2B). Western blot further confirmed these transcriptional observations (Figure 2C and Figure S2A–D).

Subsequently, we assessed the general physiological development of this mouse model. ChMKO mice exhibited no significant differences in body weight, fat/body weight, lean/body weight, or bone mineral density (BMD) (Figure S2E–H).

Next, we conducted an extensive analysis of locomotor function. Initially, exhaustive swimming tests indicated no significant difference in aerobic exercise capacity in ChMKO mice (Figure 2D). No detectable differences in post-exercise plasma LDH activity were observed between F/F and ChMKO mice under the present experimental conditions (Figure 2E). Additionally, our data revealed no measurable disparities in resting GC muscle glycogen levels between genotypes (Figure 2F). EMG recordings (Figure 2G), grip strength (Figure S2I), and gait analyses (Figure S2J–O) revealed no overt locomotor dysfunction in ChMKO mice.

Furthermore, glucose and insulin tolerance were unaltered in ChMKO mice (Figure S2P,Q). We also measured lipid concentrations in circulation and GC muscle. Although intramuscular TG content was significantly reduced in ChMKO mice (F/F: 0.0971 ± 0.0399 vs. ChMKO: 0.0608 ± 0.0332, *p* = 0.04), plasma lipid levels remained unchanged (Figure 2H and Figure S2R).

Whole-body energy metabolism was assessed using indirect calorimetry with a TSE LabMaster system in freely moving mice. Our metabolic cage readouts demonstrated that O_2_ consumption, CO_2_ production, respiratory exchange ratio (RER), and resting energy expenditure (REE) were fully comparable between F/F and ChMKO mice during both light and dark phases (Figure 2I–L).

### 3.2. Skeletal Muscle Structure and Fiber Composition Remain Unaltered in ChMKO Mice

**Figure 3:**
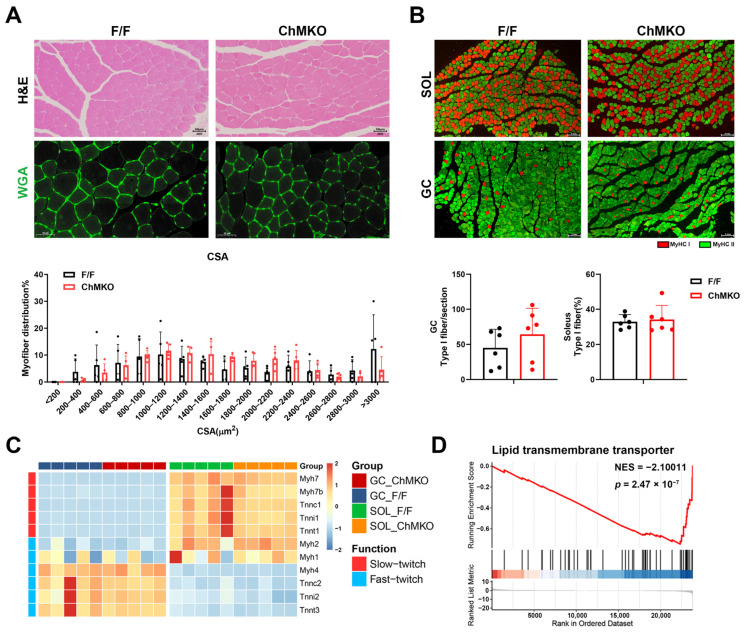
Skeletal muscle structure and fiber composition remain unaltered in ChMKO mice. (**A**) H&E and WGA staining of GC in ChMKO mice. The lower panel shows the analysis of myofiber CSA distribution. Three fields of view were captured per mouse, *n* = 5 biological replicates per group, $\eta_{p}^{2}$ = 0.01 for F/F vs. ChMKO. (**B**) Immunofluorescence staining of fast-twitch/slow-twitch myofibers in ChMKO mice. The lower panel shows the analysis of the number of slow-twitch fibers in GC and the proportion of slow-twitch myofibers in SOL, *n* = 6 biological replicates. *d* value for F/F vs. ChMKO: GC (0.60), SOL (0.22). (**C**) Heatmap of myofiber marker genes in ChMKO mice. (**D**) GSEA of DEGs derived from GC muscle; enrichment plot for the Gene Ontology (GO) term lipid transmembrane transporter activity is presented.

We next performed a comprehensive histological assessment of skeletal muscle morphology and fiber composition in ChMKO mice. Hematoxylin and eosin (H&E) and WGA staining were performed to assess skeletal muscle morphology. No obvious structural abnormalities were observed. Additionally, quantitative CSA measurements revealed that ChREBP deficiency did not alter myofiber size distribution (Figure 3A). Furthermore, fast-twitch and slow-twitch myofibers in both GC and SOL were labeled, and subsequent statistical comparisons detected no significant differences in the relative proportion of each fiber type between genotypes (Figure 3B).

RNA-seq was utilized to characterize transcriptomic profiles in GC and SOL of ChMKO mice. Principal component analysis (PCA) and unsupervised clustering of the global transcriptome revealed robust transcriptional divergence between GC and SOL tissues; by contrast, minimal differences were observed when comparing F/F control and ChMKO knockout animals (Figure S3A,B). Subsequent analysis focused on the expression of canonical myofiber markers and found no genotype-dependent changes in fast-twitch fiber markers (*Myh2*, *Myh1*, *Myh4*, *Tnnc2*, *Tnni2*, *Tnnt3*) or slow-twitch fiber markers (*Myh7*, *Myh7b*, *Tnnc1*, *Tnni1*, *Tnnt1*) (Figure 3C). Gene Set Enrichment Analysis (GSEA) analysis was performed on differentially expressed genes. No prominent enrichment was observed for core energy metabolic pathways, including glycolysis and oxidative phosphorylation (Figure S3C,D). Of note, lipid transmembrane transport pathways were significantly downregulated in ChMKO mice (Figure 3D).

### 3.3. ChREBP Overexpression Exerts Minimal Effects on Skeletal Muscle Fiber Composition

**Figure 4:**
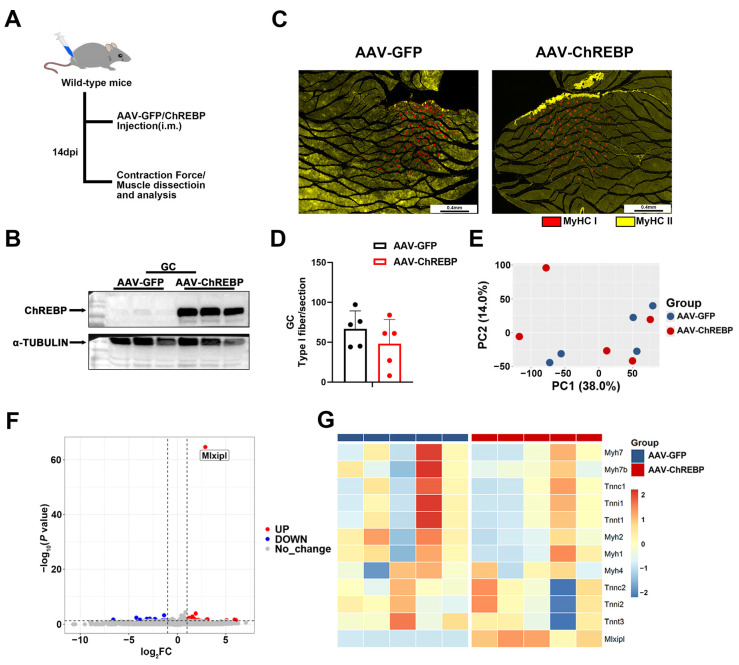
ChREBP overexpression exerts minimal effects on skeletal muscle fiber composition. (**A**) Schematic workflow of the in vivo AAV overexpression experiment. (**B**) ChREBP expression in the GC muscle was verified at 14 dpi following AAV-GFP or AAV-ChREBP injection. (**C**) Immunofluorescence staining targeting fast-twitch and slow-twitch myofibers in GC muscles post AAV injection. (**D**) Quantitative analysis of slow-twitch myofibers in GC, *n* = 5 biological replicates, *d* = 0.72. (**E**) PCA of the GC muscle at 14 dpi. (**F**) Volcano plot illustrating DEGs identified in the GC muscle at 14 dpi. Horizontal dashed line denotes the significance cutoff of *P* value; vertical dashed lines mark the log_2_FC threshold of ±1 for differentially expressed genes. (**G**) Heatmap displaying relative expression levels of canonical myofiber marker genes in GC muscle at 14 dpi.

To further elucidate the role of ChREBP in skeletal muscle, a muscle-localized overexpression mouse model was developed through intramuscular injection of AAV (Figure 4A). Successful overexpression was first confirmed at the protein level (Figure 4B). We then performed immunofluorescent staining to label fast- and slow-twitch fibers in GC muscle. Quantitative analysis detected no measurable shifts in the total count of slow-twitch fibers between AAV-GFP and ChREBP-overexpressing muscle groups (Figure 4C,D).

Next, we performed transcriptomic profiling in GC injected with AAV-ChREBP. Both PCA and DEGs analysis revealed that, despite a several-fold increase in ChREBP expression, the global transcriptional profiles of GC samples exhibited overlapping clustering among experimental groups (Figure 4E,F). Additionally, we found that ChREBP overexpression did not substantially change the transcriptional patterns associated with myofiber subtype specification (Figure 4G).

### 3.4. Muscle-Specific ChREBP Deficiency Does Not Markedly Alter Hypoxia- or Fructose-Associated Skeletal Muscle Remodeling

The skeletal muscle undergoes adaptive remodeling of myofiber types and energy metabolic function in response to physiological or pathophysiological stimuli. To address the potential role of ChREBP in skeletal muscle adaptive remodeling, we exposed ChMKO mice to a hypoxic model (simulating 5500 m altitude).

**Figure 5:**
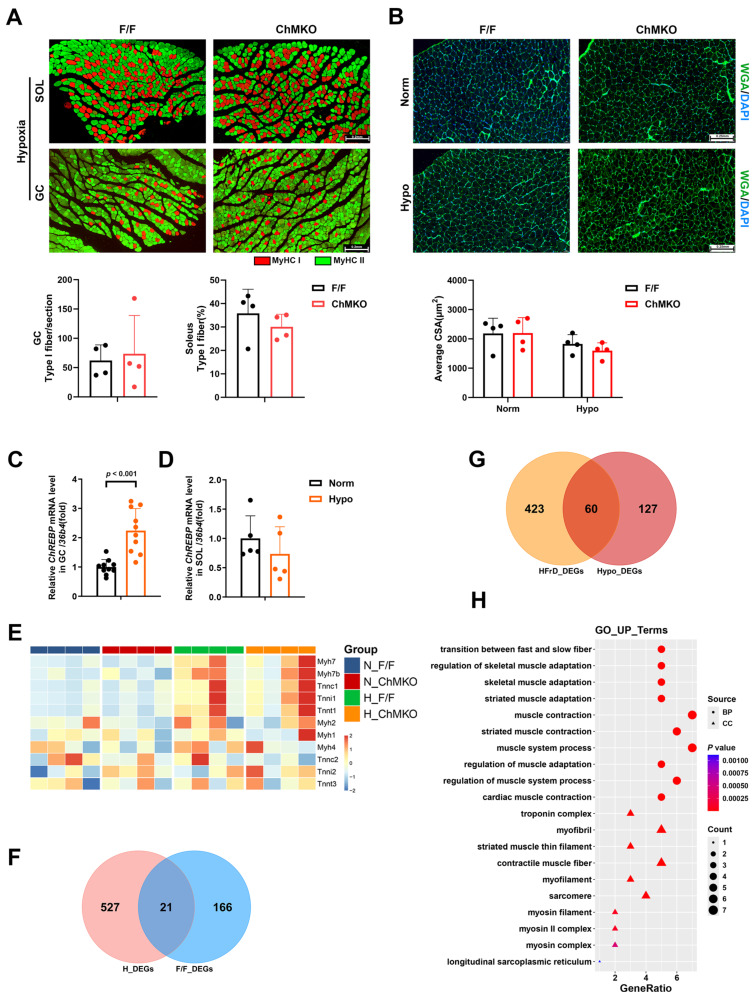
Muscle-specific ChREBP deficiency does not markedly alter hypoxia- or fructose-associated skeletal muscle remodeling. (**A**) Immunofluorescence staining of fast-twitch and slow-twitch myofiber markers in ChMKO mice after hypoxic exposure. The lower panel shows the analysis of the number of slow-twitch fibers in GC and the proportion of slow-twitch myofibers in SOL, *n* = 4 biological replicates, *d* value for F/F vs. ChMKO: GC (0.23), SOL (0.70). (**B**) WGA staining of the GC muscle in ChMKO mice after hypoxic exposure. The lower panel shows the analysis of average CSA. Three fields of view were captured per mouse, *n* = 4 biological replicates, *d* value for F/F vs. ChMKO: Norm (0.02), Hypo (0.79). (**C**,**D**) The mRNA levels of *ChREBP* in the GC (*n* = 10 biological replicates) and SOL (*n* = 5 biological replicates) muscle after 2 weeks of hypoxic exposure, 2 technical replicates per sample, Welch’s *t*-test, *d* value for Norm vs. Hypo: GC (2.28, power = 1), SOL (0.62). (**E**) Heatmap illustrating relative expression patterns of canonical myofiber marker genes in GC muscle from hypoxic ChMKO mice. (**F**) Venn diagram visualizing overlapping DEGs from two datasets: hypoxia-responsive DEGs in control littermates (F/F_DEGs) and genotype-dependent DEGs under hypoxic conditions (H_DEGs). (**G**) Venn diagram comparing DEGs derived from two datasets: hypoxic stimulation (Hypo_DEGs: Norm_F/F vs. Hypo_F/F) and high-fructose diet challenge (HFrD_DEGs: Norm_F/F vs. HFrD_F/F). (**H**) GO enrichment analysis of the 18 commonly upregulated DEGs in HFrD_DEGs and Hypo_DEGs. The top 10 most significantly enriched pathways in biological processes and cellular component are shown.

Initially, we investigated body composition in response to hypoxic exposure. Compared with normoxic controls, hypoxia lowered body weight (*p* < 0.001), fat/body weight (F/F: *p* = 0.001; ChMKO: *p* = 0.002) and BMD (*p* = 0.003), but raised lean/body weight (F/F: *p* = 0.001; ChMKO: *p* = 0.002) in both genotypes. No significant intergenotype differences were observed between ChMKO and F/F littermates (Figure S4A–D). Meanwhile, slow-twitch fiber counts in GC and fractional proportion in SOL were indistinguishable between ChMKO and controls (Figure 5A). Consistently, the mean CSA of myofibers did not differ between the two mouse lines (Figure 5B).

Subsequently, in wild-type mice, we investigated the *ChREBP* mRNA levels across various murine tissues following exposure to hypoxic conditions. Our findings showed that hypoxia robustly increased *ChREBP* mRNA in small intestine and GC (both *p* < 0.001), accompanied by elevated intestinal *Pklr* (*p* < 0.001) (Figure 5C and Figure S4E), while *ChREBP* in liver and SOL, as well as hepatic *Pklr*, remained unchanged (Figure 5D and Figure S4F).

RNA-seq analysis revealed upregulated expression of slow-twitch myofiber markers in GC upon hypoxic exposure. However, transcriptomic patterns did not differ substantially between F/F and ChMKO mice (Figure 5E). Venn diagram comparison revealed merely 21 overlapping DEGs between two datasets: hypoxia-responsive DEGs in control mice (F/F_DEGs: Norm_F/F vs. Hypo_F/F) and genotype-dependent DEGs under hypoxic conditions (H_DEGs: Hypo_F/F vs. Hypo_ChMKO) (Figure 5F).

We then conducted a combined enrichment analysis on hypoxia-induced F/F_DEGs and ChMKO_DEGs (Norm_ChMKO vs. Hypo_ChMKO). The 24 shared upregulated genes were predominantly enriched in gene sets associated with skeletal muscle structure and function (Figure S4G,H). In contrast, enrichment analysis of DEGs exclusive to hypoxic ChMKO mice (88 upregulated and 339 downregulated) revealed that these genes were primarily enriched in metabolic processes, with minimal involvement in skeletal muscle-related pathways (Figure S4I,J).

We further explored the role of ChREBP in skeletal muscle remodeling triggered by high-fructose diet challenge. Wild-type mice were fed a HFrD for 14 days to assess ChREBP activation. We found that in the small intestine, *ChREBP-α* (*p* = 0.003), *ChREBP-β* (*p* = 0.01) and its downstream target gene *Pklr* were significantly activated (*p* < 0.001) (Figure S5A). However, in GC, *ChREBP* expression remained unaltered (Figure S5B). Additionally, quantitative analysis revealed that the HFrD induced modest fluctuations in fast-twitch myofiber markers, while leaving slow-twitch fibers largely unaffected (Figure S5C,D).

We subsequently extended the HFrD intervention to three months, resulting in significant reductions in both body weight and fat/body weight, as well as an increase in lean/body weight (*p* < 0.001) (Figure S5E). Transcriptomic analysis indicated that muscle-specific ChREBP ablation did not induce obvious transcriptional separation between ChMKO and F/F groups (Figure S5F). Although slow-twitch fiber markers were increased in skeletal muscle, muscle ChREBP expression remained stable (Figure S5G,H).

Finally, we compared the skeletal muscle transcriptional alterations induced by hypoxic exposure and long-term high-fructose feeding. RNA-seq profiling of GC muscles from F/F mice identified 483 DEGs responsive to long-term high-fructose feeding (HFrD_DEGs) and 187 hypoxia-inducible DEGs (Hypo_DEGs). Notably, Venn diagram intersection revealed a set of 60 overlapping DEGs altered under both experimental stress models (Figure 5G). Among these, 18 commonly upregulated DEGs were predominantly enriched in pathways governing skeletal muscle structure and composition (Figure 5H and Figure S5I).

### 3.5. ChREBP Overexpression Promotes Myogenic Differentiation in C2C12 Cells In Vitro

**Figure 6:**
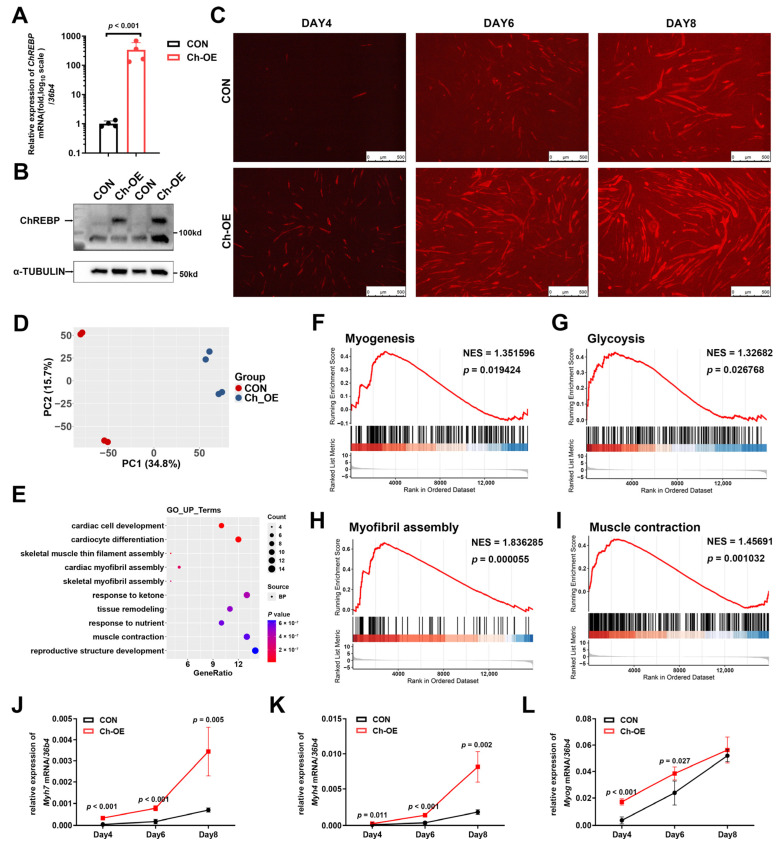
ChREBP overexpression promotes myogenic differentiation in C2C12 cells in vitro. (**A**,**B**) Validation of ChREBP expression in the Ch-OE cell line by qRT-PCR and Western blot assays, *n* = 4 biological replicates (independent cell culture wells), 2 technical replicates per sample, Welch’s *t*-test, *d* = 1.85, power = 0.98. (**C**) Immunofluorescent labeling of MHC in CON and Ch-OE cells on day 4, 6, and 8 of myogenic differentiation. (**D**) PCA of gene expression in Ch-OE cells on day 7 of myogenic differentiation. (**E**) GO enrichment analysis of 224 upregulated DEGs in Ch-OE cells. The top 10 most significantly enriched pathways in biological processes are shown. (**F**–**I**) GSEA of DEGs in Ch-OE cells on day 7 of myogenic differentiation induction, showing enrichment plots for Hallmark gene sets (**F**,**G**) and GO gene sets (**H**,**I**). (**J**–**L**) The mRNA levels of *Myh7*, *Myh4*, and *Myog* on Day 4, 6, and 8 of myogenic differentiation, *n* = 4 biological replicates, 2 technical replicates per sample. Data were analyzed using unpaired two-tailed Student’s *t*-test, with Welch’s correction applied when variances were unequal. Labeled *p* values denote CON vs. Ch-OE comparisons within the same time point, *d* values for CON vs. Ch-OE: *Myh7* (5.96, 5.69, 3.36), *Myh4* (2.30, 5.76, 4.07), *Myog* (5.66, 2.03, 0.56) at Day 4, 6 and 8, respectively, power = 1.

We further investigated the function of ChREBP in post-injury muscle repair and regeneration. The C2C12 cell line is a well-established model for the in vitro examination of myogenic differentiation and the structural function of myotubes. We found that ChREBP expression tended to increase during the early stage of C2C12 myogenic differentiation (*p* = 0.008) (Figure S6A,B). Given the relatively low endogenous expression of ChREBP in C2C12 cells, we therefore established a ChREBP-overexpressing (Ch-OE) cell line using lentiviral vectors. Successful overexpression was confirmed at both mRNA and protein levels (*p* < 0.001) (Figure 6A,B). We found that myogenic differentiation was markedly accelerated in Ch-OE cells relative to empty-vector controls (Figure 6C).

Consequently, RNA-seq analysis demonstrated a clear transcriptional separation between the control and Ch-OE groups (Figure 6D). GO enrichment analysis indicated that the upregulated DEGs were predominantly associated with pathways involved in skeletal muscle development and function (Figure 6E and Figure S6C). GSEA revealed significant activation of pathways related to myogenesis, glycolysis and muscle structure (Figure 6F–I). Furthermore, we quantified mRNA levels of canonical myofiber marker genes at day 4, 6 and 8 post differentiation induction. The results indicated that the mRNA expression levels of *Myh4* and *Myh7* in Ch-OE cells were significantly elevated compared to control cells, and this difference increased progressively during differentiation (Figure 6J,K). Moreover, the expression of *Myl1*, *Myl2*, *Myl3*, *Tnnc1*, *Tnni1*, *Tnnt1*, *Tnnc2*, *Tnni2* and *Tnnt3* was also upregulated on day 4 and 6 (Figure S6D).

To explore the molecular mechanism underlying ChREBP-mediated promotion of myogenic differentiation, we analyzed the expression of core myogenic regulators including *Pax7*, *Myod1*, and *Myog*. The difference in *Myog* mRNA expression between groups was most pronounced at the early differentiation stage and gradually attenuated thereafter (Figure 6L). Consistently, MYOG protein abundance was already markedly increased in proliferating Ch-OE myoblasts before differentiation was initiated (Figure S6E,F).

### 3.6. Genetic Ablation of ChREBP Exerts Negligible Impacts on Skeletal Muscle Injury Regeneration

**Figure 7:**
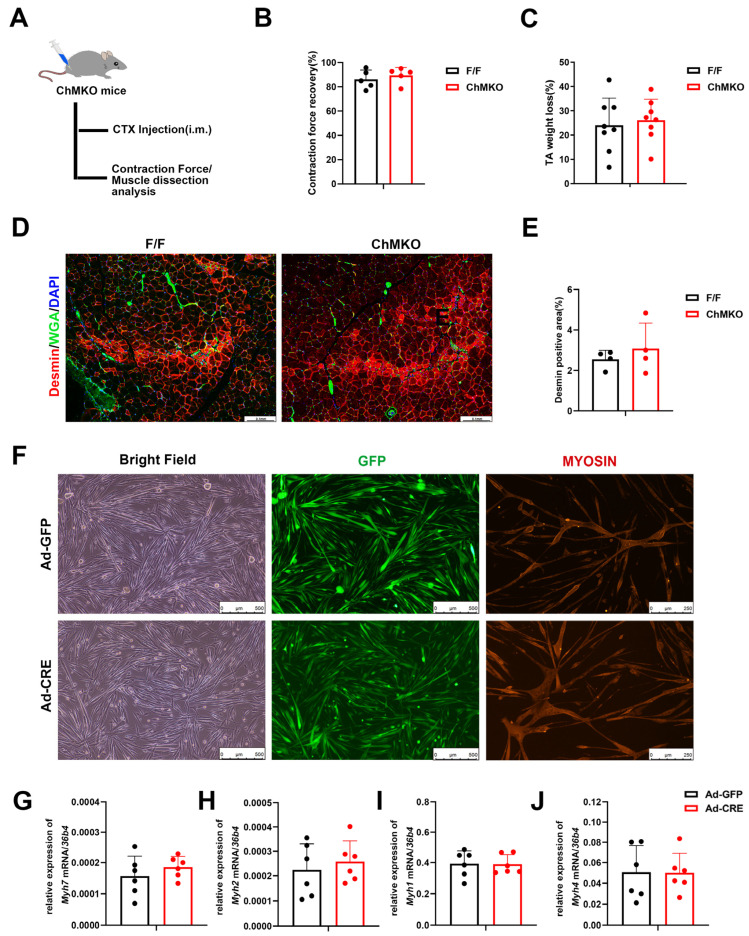
Genetic ablation of ChREBP exerts negligible impacts on skeletal muscle injury regeneration. (**A**) Schematic diagram depicting the workflow for constructing TA muscle injury models in ChMKO mice via intramuscular CTX injection. (**B**) EMG analysis of the TA muscle in ChMKO mice at 14 dpi, *n* = 5 biological replicates, *d* = 0.47. (**C**) Analysis of TA muscle weight loss in ChMKO mice at 14 dpi, normalized to the contralateral control leg from the same mouse, *n* = 8 biological replicates, *d* = 0.21. (**D**) Desmin and WGA staining analysis of the TA muscle at 14 dpi. (**E**) Morphometric quantification of the desmin-positive regenerative area on TA cross sections, *n* = 4 biological replicates, *d* = 0.55. (**F**) Morphological analyses and immunofluorescence staining of MHC were performed to evaluate myogenic differentiation capacity. (**G**–**J**) The mRNA levels of *Myh7*, *Myh2*, *Myh1* and *Myh4* in myotubes derived from MuSCs after ChREBP knockout, *n* = 6 biological replicates, 2 technical replicates per sample, *d* values for Ad-GFP vs. Ad-CRE: *Myh7* (0.67), *Myh2* (0.35), *Myh1* (0.05), *Myh4* (0.02).

To investigate the role of ChREBP in myogenesis in vivo, we established an acute skeletal muscle injury model by administering CTX into the TA of ChMKO mice (Figure 7A). EMG signals were recorded from the TA muscles at 14 dpi. Quantitative analysis revealed no significant differences in the recovery of contractile function relative to the uninjured contralateral TA between genotypes (Figure 7B). We further evaluated post-injury muscle atrophy at 14 dpi, and the extent of muscle weight loss was comparable between ChMKO and F/F littermates (Figure 7C).

We used desmin and WGA to label regenerating myofibers and the sarcolemma, respectively. Morphometric analysis revealed no obvious differences in the area of desmin-positive regenerating tissue among groups (Figure 7D,E).

We next analyzed the potential functions of ChREBP in MuSCs with GSE59272 dataset from the Gene Expression Omnibus (GEO) database [37], which comprises microarray expression profiles of mouse primary MuSCs at distinct differentiation stages. Our analysis showed that ChREBP expression gradually rises alongside progressive differentiation of activated satellite cells (Figure S7A). Primary MuSCs isolated from ChREBP-Flox mice were subsequently transduced with Cre-expressing adenovirus and cultured in medium supplemented with 2% horse serum to induce differentiation. Immunoblot analysis confirmed efficient ChREBP knockout in differentiated myotubes (Figure S7B). Additional immunofluorescent staining and morphometric analysis demonstrated similar myogenic dynamics and cell fusion characteristics of MuSCs between groups (Figure 7F and Figure S7C).

qRT-PCR confirmed robust downregulation of ChREBP expression following Ad-Cre treatment (*p* < 0.001) (Figure S7D). The core myogenic transcription factors *Pax7*, *Myod1* and *Myog* showed no significant differences between groups (Figure S7E–G). Similarly, mRNA levels of pan-myofiber markers (*Myh7*, *Myh2*, *Myh1* and *Myh4*) remained unaltered after ChREBP ablation (Figure 7G–J).

### 3.7. Overexpression of ChREBP Enhances Skeletal Muscle Regeneration In Vivo

**Figure 8:**
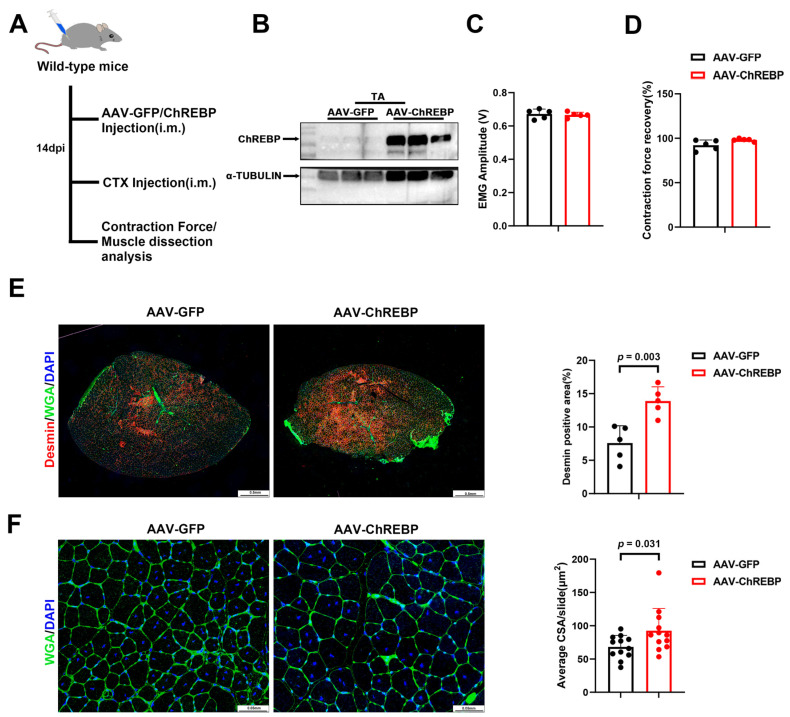
Overexpression of ChREBP enhances skeletal muscle regeneration in vivo. (**A**) Schematic of experimental design: local AAV-mediated ChREBP overexpression in mouse TA muscle followed by CTX-induced acute muscle injury. (**B**) ChREBP expression in the TA muscle was verified at 14 dpi of AAV-GFP or AAV-ChREBP. (**C**) EMG analysis of the TA muscle at 14 days post AAV injection, *n* = 5 biological replicates, *d* = 0.26. (**D**) EMG analysis of contractile strength recovery in the TA muscle at 14 days post CTX injection, *n* = 5 biological replicates, *d* = 1.39, power = 0.86. (**E**) Desmin staining of TA muscle at 7 days post CTX injection; the right panel shows the quantification of Desmin-positive areas, *n* = 5 biological replicates, unpaired two-tailed Student’s *t*-test, *d* = 2.65, power = 1. (**F**) WGA staining of TA muscle at 14 days post CTX injection; the right panel shows CSA analysis of new myofibers with central nuclei. A total of 12 fields were captured from 5 biological replicates per group, Welch’s *t*-test, *d* = 0.92, power = 0.89.

Consequently, we further explored the in vivo effects of ChREBP overexpression on skeletal muscle repair after acute injury (Figure 8A). Successful protein overexpression was first validated (Figure 8B). ChREBP overexpression caused no substantial changes in basal muscle contractile function (Figure 8C). Of note, at 14 dpi, EMG analysis revealed a trend toward improved contractile strength recovery in the TA muscle of the ChREBP-overexpressing group compared with empty-vector controls (AAV-GFP: 92.24 ± 5.62 vs. AAV-ChREBP: 98.14 ± 1.61, *p* = 0.084) (Figure 8D).

Quantitative analysis showed significantly larger desmin-positive regenerating areas in AAV-ChREBP-injected TA relative to AAV-GFP controls at 7 days post CTX injury (*p* = 0.003) (Figure 8E). We further measured the CSA of newly formed centronuclear myofibers at 14 days post CTX injury and observed a larger mean myofiber size in the ChREBP-overexpressing group (*p* = 0.031) (Figure 8F). We additionally measured intramuscular IL-13, TNF-α and IFN-γ levels at 7 dpi. No detectable differences in these inflammatory cytokines were observed between F/F and ChMKO mice (Figure S8A–C).

## 4. Discussion

The present study explored the roles of ChREBP in skeletal muscle physiology, adaptive remodeling and injury repair. Our findings demonstrate that ChREBP exerts distinct functions under different biological states: its genetic ablation has little impact on basal muscle homeostasis, as well as on myofiber remodeling induced by hypoxia or high-fructose challenges. In contrast, ChREBP promotes myogenic differentiation and facilitates the regeneration of damaged skeletal muscle.

### 4.1. ChREBP Deficiency Exerts Negligible Effects on Basal Muscle Homeostasis and Adaptive Remodeling

Previous research has identified several regulators involved in the coordination between myofiber phenotype and muscle metabolic homeostasis, including transcription factor sex-determining region Y-box 6 (SOX6), nuclear receptors including peroxisome proliferator-activated receptors (PPARs) and estrogen-related receptors (ERRs), myocyte enhancer factor 2C (MEF2C), as well as co-regulators such as peroxisome proliferator-activated receptor gamma coactivator 1α (PGC1α), nuclear receptor corepressor 1 (NCoR1), histone deacetylases (HDACs), mixed-lineage leukemia 4 (MLL4), and calcineurin/nuclear factor of activated T cells (NFAT) [38,39,40,41,42,43]. Nevertheless, whether ChREBP participates in the regulation of skeletal muscle fiber composition and metabolic status remains unclarified.

In the present study, we originally hypothesized that ChREBP would be involved in multiple physiological processes of skeletal muscle. Contrary to our expectation, studies with muscle-specific knockout mice and local AAV-driven overexpression demonstrated that ChREBP deletion did not lead to measurable alterations in exercise performance, general metabolic function, or myofiber composition under the current experimental settings. In addition, ChREBP ablation had minimal effects on hypoxia- or HFrD-triggered myofiber remodeling. Taken together, these findings indicate that ChREBP is largely dispensable for maintaining basal muscle homeostasis and stress-induced adaptive remodeling.

The mild phenotype of ChMKO mice is most likely caused by functional compensation by another glucose-sensing transcription factor, MondoA (also referred to as MLX-interacting protein, MLXIP). Both MondoA and ChREBP are members of the bHLH/LZ family of transcription factors, which are capable of forming heterodimeric complexes with Max-like protein X (MLX) to modulate gene expression in response to glucose and other nutrients [22,24,44]. Previous studies have shown that MondoA regulates genes related to glucose metabolism, glycogen synthesis and insulin signaling, and is closely linked to the pathogenesis and progression of metabolic disorders including obesity, insulin resistance and type 2 diabetes mellitus [44,45]. In contrast, ChREBP responds to carbohydrate consumption and controls the expression of genes related to glycolysis and de novo lipogenesis. Despite shared structural homology and overlapping nutrient-sensing functions, MondoA and ChREBP execute distinct physiological roles and target different downstream gene networks. Existing literature documents predominant MondoA expression and function in skeletal muscle, yet ChREBP is also abundantly expressed within this tissue. The complicated crosstalk between MondoA and ChREBP, as well as their respective downstream transcriptional cascades, has not been fully characterized to date.

Our transcriptomic analyses further revealed that hypoxic exposure upregulated slow-twitch myofiber signature genes, a transcriptional pattern highly similar to that induced by long-term HFrD feeding. Shared DEGs identified under hypoxic and high-fructose conditions were primarily enriched in pathways governing myofiber type switching, indicating potential molecular crosstalk underlying skeletal muscle adaptive responses to these two distinct stress stimuli. However, a direct comparison of fructose catabolism and hypoxia-driven metabolic rewiring is beyond the scope of the current study. Therefore, the putative molecular association between hypoxic stress and dietary metabolic stress remains a hypothesis-generating observation and requires comprehensive experimental validation in dedicated follow-up investigations.

Since hypoxia and HFrD were primarily applied as stress stimuli for myofiber adaptive remodeling in this study, dynamic alterations of fructose metabolism in skeletal muscle were not specifically assessed in our experimental system. Our RNA-seq datasets illustrated that chronic high-fructose feeding did not trigger obvious transcriptional shifts in muscle *ChREBP* or *SLC2A5* (Solute carrier family 2 member 5, GLUT5). Although transcriptomic changes alone cannot rule out potential functional variations in fructose transport and metabolism, these negative transcriptional outcomes prompted us to further focus on the regulatory effects of ChREBP on myofiber phenotypic remodeling. Even so, the lack of systematic protein and metabolite validation of core fructose catabolic enzymes restricts our capacity to fully delineate muscle fructose metabolic networks under ChREBP deficiency. Subsequent research will incorporate the detection of rate-limiting metabolic enzymes and untargeted metabolomic profiling to accurately decipher time-dependent dynamic shifts in fructose metabolism upon muscle-specific ChREBP ablation.

### 4.2. ChREBP Appears to Facilitate Myogenic Differentiation and Promote Skeletal Muscle Regeneration

The plasticity of skeletal muscle is further demonstrated by its remarkable regenerative capacity. Notably, we identified a pro-regenerative role of ChREBP in myogenic differentiation. Specifically, gain-of-function experiments in C2C12 myoblasts demonstrated that ChREBP overexpression substantially promotes myogenic differentiation, accompanied by elevated expression of *Myh7*, *Myh4*, and multiple troponin isoforms. MYOG was significantly upregulated at the early stage of differentiation. Consistently, local AAV-mediated ChREBP overexpression enhanced histological indices of regeneration, including desmin-positive regenerative area and the cross-sectional size of newly formed myofibers following CTX-induced injury.

Muscle repair after injury is a highly orchestrated process involving myofiber degeneration, inflammatory infiltration, de novo myogenesis and tissue maturation, which relies on the coordinated crosstalk among MuSCs, immune cells and stromal cells [46]. Given that MuSCs serve as the primary cellular source for muscle regeneration, we further examined the intrinsic function of ChREBP in primary MuSCs. Our analysis of the GSE59272 dataset demonstrated a gradual increase in ChREBP levels during myogenic differentiation. It is well established that satellite cell activation is accompanied by increased energy demand and profound metabolic remodeling, including enhanced glycolysis [47]. In line with this notion, GSEA of cellular experiments demonstrated that higher ChREBP levels correlate with strengthened glycolytic activity in C2C12 cells. Despite the supportive correlative evidence linking ChREBP to myogenic metabolic reprogramming, our loss-of-function results suggest that endogenous ChREBP is not strictly required for MuSCs differentiation under basal culture conditions. Notably, primary MuSCs isolated via enzymatic digestion in this in vitro system cannot fully recapitulate the transition from quiescent to activated states that occurs in vivo. Therefore, whether ChREBP participates in MuSCs activation and subsequent myogenic differentiation during in vivo regeneration remains speculative and requires further validation.

The progression of myogenic differentiation is tightly governed by the sequential activation and repression of core transcription factors, including *Pax7*, *Myod1* and *Myog* [48]. *Pax7* maintains the undifferentiated state of myoblasts and declines throughout differentiation; *Myod1* drives myogenic commitment at the early stage, while *Myog* functions in mid-to-late differentiation to facilitate cell fusion and the expression of muscle structural proteins such as troponin. We detected no obvious changes in *Pax7* and *Myod1* expression in ChREBP-overexpressing C2C12 cells. In contrast, differential *Myog* expression was observed as early as day 4 after differentiation induction. The intergroup difference in *Myog* mRNA was most prominent in early differentiation and gradually attenuated over time. Consistent with the transcriptional pattern, MYOG protein levels appeared to be elevated in ChREBP-overexpressing myoblasts even during the proliferative stage. Collectively, these observations suggest that ChREBP overexpression has minimal impacts on *Pax7*-associated myoblast proliferation and *Myod1*-guided differentiation initiation. Instead, the accelerated cell fusion and increased *Myog* expression raise the possibility that *Myog* serves as a candidate downstream mediator of ChREBP during myogenic progression.

Our AAV-mediated overexpression model further confirmed the positive regulatory effect of ChREBP on muscle injury repair. As a preliminary approach to explore whether ChREBP governs skeletal muscle regeneration, the AAV-mediated overexpression system permits rapid and straightforward characterization of its overall effects. However, this non-cell-specific manipulation cannot distinguish which cell population mediates the pro-regenerative actions of ChREBP. Since immune and inflammatory signaling are critical modulators of MuSCs expansion [36], we further examined whether ChREBP overexpression modulates inflammatory responses during muscle regeneration. Our results showed that CTX injury robustly induced the expression of IL-13, TNF-α and IFN-γ in TA muscle, yet the injury-triggered inflammatory responses were comparable between control and ChREBP-overexpressing groups. These findings exclude inflammatory cytokine signaling as a major downstream mechanism of ChREBP-mediated muscle regeneration.

In addition to immune factors, various cytokines and growth factors derived from mature muscle fibers are also recognized as playing pivotal roles in regulating muscle repair and growth, such as myostatin, transforming growth factor-β (TGF-β), Dickkopf-related protein 3 (DKK3), and insulin-like growth factor-1 (IGF-1) [49,50,51]. Notably, our ChMKO mice, with ChREBP specifically deleted in differentiated myofibers, retained normal regenerative capacity after acute CTX injury, indicating that ChREBP in mature myofibers is unlikely to be a key mediator of paracrine-dependent muscle repair.

Taken together, our results imply that MuSCs are likely the main cellular target through which ChREBP promotes skeletal muscle regeneration. However, two major gaps remain to be addressed. First, the temporal expression pattern of endogenous ChREBP in MuSCs throughout the course of muscle regeneration has not been comprehensively profiled. Second, conclusive evidence defining the biological function of ChREBP exclusively within MuSCs is still missing; this underscores the need to generate and phenotype MuSCs-specific knockout and overexpression mouse models.

Furthermore, as a canonical transcription factor, mapping ChREBP’s key downstream target genes and delineating its transcriptional regulatory network are indispensable to unravel the molecular basis for ChREBP-governed myogenesis and muscle regeneration. Our present results pinpoint *Myog* as a plausible downstream effector mediating ChREBP-dependent myogenic differentiation; further rigorous investigations encompassing transcriptional binding characterization and hierarchical functional verification are essential to consolidate this regulatory axis. Nevertheless, such comprehensive mechanistic validation falls beyond the scope of the present study, representing one of the primary limitations that preclude full clarification of their precise regulatory interplay.

### 4.3. Methodological and Experimental Limitations

Several methodological and technical limitations should be acknowledged in the present work. The scope of in vivo functional evaluation was constrained by the selected readouts and animal strains. We assessed muscle function via swimming endurance, grip strength, gait kinematics and systemic metabolic phenotyping. Nonetheless, these measurements cannot recapitulate the full spectrum of myofiber contractile properties and intrinsic physiological characteristics. Several complementary assays, including treadmill endurance testing, ex vivo muscle force recording, fatigue tolerance assessment and mitochondrial respiratory capacity profiling, were not included in the current experimental setup. Incorporating these approaches in future work will support a more comprehensive interpretation of skeletal muscle functional phenotypes under metabolic or hypoxic stress.

Additionally, the MCK-Cre mouse model used in this study has inherent caveats. While this strain is widely employed for skeletal muscle-specific gene manipulation, Cre-mediated recombination is also present in cardiac tissue. Given that ChREBP expression was reduced in the hearts of ChMKO mice, we cannot entirely rule out the possibility that altered cardiac function may partially contribute to the observed physiological phenotypes. The human skeletal actin Cre (HSA-Cre) line, which drives highly specific recombination in skeletal muscle with minimal activity in the heart [39,52], would be a more optimal choice to eliminate cardiac interference on exercise performance. Addressing these limitations in follow-up work will further clarify the ChREBP-dependent regulatory mechanisms governing skeletal muscle physiology and stress adaptation.

This study was statistically powered to detect biologically relevant phenotypic changes in core experimental outcomes. Nevertheless, we acknowledge that the limited sample size may hinder the detection of subtle phenotypic variations. Notably, two analyses exhibited large effect sizes without reaching statistical significance. Among these, the trending recovery of TA muscle contractile strength possessed adequate post hoc statistical power and was therefore presented in the main text. Only the index of contralateral gait coordination showed marginal insufficiency in post hoc power. Given the inherent variability of mouse gait behavior and uniformly negative outcomes across other gait parameters, this minor discrepancy likely originated from individual animal variation and does not affect the primary conclusions of the present study. Expanding sample size in future work will improve the robustness and reproducibility of relevant phenotypic observations.

This work also presents methodological limitations in transcriptomic analysis. DEGs were identified based on uncorrected *p* values (*p* < 0.05) and |log_2_FC| ≥ 1 without FDR adjustment. This exploratory screening strategy was intentionally adopted because stringent FDR filtering markedly reduced the candidate gene pool and compromised the stability and reliability of downstream functional enrichment analyses, which was the major objective of our RNA-seq profiling. Even so, we acknowledge that uncorrected statistical thresholds may introduce a moderate risk of false-positive genes and lower analytical stringency, warranting cautious interpretation of the present transcriptomic results. Future studies with larger sample sizes, FDR-adjusted statistics, and independent experimental validation will help refine the ChREBP-regulated transcriptional networks and yield more robust transcriptomic signatures in skeletal muscle.

## 5. Conclusions

Taken together, the integrated findings from our multiple experimental models indicate that genetic ablation of ChREBP exerts minor modulatory effects on skeletal muscle function and myofiber subtype composition. Consistently, loss of endogenous ChREBP does not appear to alter skeletal muscle adaptive remodeling triggered by hypoxic exposure or long-term high-fructose feeding. Beyond these negative phenotypic observations, our cellular and in vivo functional studies confirm a physiological role for ChREBP in skeletal muscle: upregulated ChREBP accelerates myogenic differentiation and moderately enhances regenerative capacity following acute muscle injury. The present study hence illustrates that ChREBP may act as one beneficial modulator supporting muscle damage repair, and these findings provide novel experimental clues for future translational research targeting skeletal muscle injury treatment and functional rehabilitation.

## Data Availability

The RNA-seq datasets generated in this study are accessible from GEO with accession number GSE319093. Data supporting the findings of this study are available within the article and its Supplementary Information files or from the corresponding author upon reasonable request.

## Supplementary Materials

The following supporting information can be downloaded at: https://www.mdpi.com/article/10.3390/nu18122012/s1, Figure S1: Analysis of ChREBP Expression in Different Skeletal Muscle; Figure S2: Phenotypic Analysis of ChMKO Mice; Figure S3: RNA-seq Analysis of the GC and SOL Muscles in ChMKO Mice; Figure S4: Effects of Hypoxic Exposure on Skeletal Muscle Phenotype and ChREBP Expression; Figure S5: Analysis of Chronic High-Fructose Diet on Skeletal Muscle Phenotype and ChREBP Expression; Figure S6: ChREBP Enhances Myogenic Differentiation in C2C12 Cells; Figure S7: Analysis of the Function of ChREBP in Primary Skeletal Muscle Satellite Cells; Figure S8: Detection of Inflammatory Cytokines in a ChREBP-Overexpressing Skeletal Muscle Injury Model; Table S1: Primer information for qRT-PCR analysis; Table S2: Number of DEGs.

## Abbreviations

The following abbreviations are used in this manuscript:

AAV	Adeno-associated virus
Ad-Cre	Adenovirus-Cre
ANOVA	Analysis of variance
BCA	Bicinchoninic acid
BKY	Two-stage linear step-up Benjamini–Krieger–Yekutieli FDR correction
BMD	Bone mineral density
bFGF	Basic fibroblast growth factor
bHLH/LZ	Basic helix–loop–helix/leucine zipper
ChMKO	ChREBP muscle-specific knockout
ChREBP	Carbohydrate response element-binding protein
Ch-OE	ChREBP-overexpressing cell line
CMV	Cytomegalovirus
Cre	Cre recombinase
CSA	Cross-sectional area
CTX	Cardiotoxin
DEGs	Differentially expressed genes
DIA	Diaphragm
DMEM	Dulbecco’s modified eagle medium
DKK3	Dickkopf-related protein 3
dpi	Days post-injection
EDL	Extensor digitorum longus
EMG	Electromyography
ERRs	Estrogen-related receptors
F/F	ChREBP-floxed control mice
FBS	Fetal bovine serum
FDR	Benjamini–Hochberg false discovery rate
GC	Gastrocnemius
GEO	Gene expression omnibus
GFP	Green fluorescent protein
GLUT5	Glucose transporter type 5
GO	Gene ontology
GSEA	Gene set enrichment analysis
H&E	Hematoxylin and eosin
HFrD	High-fructose diet
HDACs	Histone deacetylases
HSA-Cre	human skeletal actin Cre mice
IFN-γ	Interferon-γ
IGF-1	Insulin-like growth factor-1
IL-13	Interleukin-13
IL-1α	Interleukin-1α
ITT	Insulin tolerance test
Khk	Ketohexokinase
LDH	Lactate dehydrogenase
loxP	Locus of X-over P1
MCK	Muscle creatine kinase
MEF2C	Myocyte enhancer factor 2c
MHC	Myosin heavy chain
MLL4	Mixed lineage leukemia 4
MLX	Max-like protein X
MLXIP	MLX-interacting protein, MondoA
MLXIPL	MLX-interacting protein-like
MuSCs	Muscle satellite cells
Myh1	Myosin heavy chain 1
Myh2	Myosin heavy chain 2
Myh4	Myosin heavy chain 4
Myh7	Myosin heavy chain 7
Myl1	Myosin light chain 1
Myl2	Myosin light chain 2
Myl3	Myosin light chain 3
Myod1	Myoblast determination protein 1
Myog	Myogenin
NCoR1	Nuclear receptor corepressor 1
NFAT	Nuclear factor of activated T cells
NLS	Nuclear localization signal
OGTT	Oral glucose tolerance test
Pax7	Paired box 7
PBS	Phosphate-buffered saline
PCA	Principal component analysis
PFU	Plaque-forming unit
PGC1α	Peroxisome proliferator-activated receptor γ coactivator 1-α
Pklr	Pyruvate kinase l/r
PPARs	Peroxisome proliferator-activated receptors
PVDF	Polyvinylidene difluoride
qRT-PCR	Quantitative real-time polymerase chain reaction
REE	Resting energy expenditure
RER	Respiratory exchange ratio
RNA-seq	RNA sequencing
SD	Standard deviation
SDS-PAGE	Sodium dodecyl sulfate-polyacrylamide gel electrophoresis
SLC2A5	Solute carrier family 2 member 5
SOL	Soleus
SOX6	SRY-box transcription factor 6
SREBP	Sterol regulatory element-binding protein
TA	Tibialis anterior
TC	Total cholesterol
TG	Triglyceride
TGF-β	Transforming growth factor-β
TNF-α	Tumor necrosis factor-α
Tnnc1	Troponin c1, slow skeletal and cardiac type
Tnnc2	Troponin c2, fast skeletal type
Tnni1	Troponin i1, slow skeletal type
Tnni2	Troponin i2, fast skeletal type
Tnnt1	Troponin t1, slow skeletal type
Tnnt3	Troponin t3, fast skeletal type
WGA	Wheat germ agglutinin
