# Supplementary material for: ChREBP Is Dispensable for Myofiber Type Switch but Promotes Skeletal Muscle Regeneration
Source: Nutrients. 2026 Jun 21;18(12):2012. doi: 10.3390/nu18122012 (PMC13305853; doi:10.3390/nu18122012)
Supplement: Supplementary file 1 [file nutrients-18-02012-s001.zip › Supplementary figures legend-Proof-Corrected.pdf]

Figure S1. Analysis of ChREBP Expression in Different Skeletal Muscle.

The mRNA of *ChREBP-α* was detected from the GC, TA, EDL, SOL, and DIA muscles of 8-week-old male C57BL/6 mice, n = 10 biological replicates, 2 technical replicates per sample. Kruskal-Wallis H test, $\varepsilon^2 = 0.002$.

Figure S2. Phenotypic Analysis of ChMKO Mice.

(A-D) Validation of ChREBP knockout efficiency and tissue specificity in liver, intestine, TA muscle, and heart by Western blot analysis.

(E) Body weight growth curves of F/F and ChMKO mice, n = 10 biological replicates.

(F-H) Comparison of lean/body weight, fat /body weight, and BMD, F/F, n = 14; ChMKO, n = 15 biological replicates, d value for F/F vs. ChMKO: 0.36, 0.16, 0.13, respectively.

(I) Grip strength was assessed in ChMKO mice. Each mouse was measured three times, and the mean value was calculated and normalized to body weight, n = 5 biological replicates, *d* = 0.30.

(J-O) Gait analysis was performed in ChMKO mice to assess stride cycle, overall speed, stance phase, average swing speed, contralateral coordination, and duty cycle. All parameters were quantified for the right hind limb (RH), n = 5 biological replicates. Stride cycle: Duration from heel strike to the subsequent heel strike of the right hind paw, *d* = 0.52. Overall speed: Total distance traveled divided by total walking duration, *d* = 0.61. Stance phase: Period during which the right hind paw contacts the ground within a stride cycle (complementary to swing phase), *d* = 0.43. Swing speed: Stride length of the right hind limb divided by its swing time duration, *d* = 0.27. Contralateral coordination: Ratio of the swing or stance duration of the right hind paw (RH) to the stride cycle of the left front paw (LF), *p* = 0.113, *d* = 1.13, power = 0.76. Duty cycle: Percentage of total time that the right hind paw remains in contact with the ground, *d* = 0.19.

(P) OGTT in ChMKO mice following administration of 2.0 g/kg body weight glucose, F/F, n = 14; ChMKO, n = 13 biological replicates, *d* value for F/F vs. ChMKO: 0min (0.16), 15min (0.34), 30min (0.63), 60min (0.46), 120min (0.56).

(Q) ITT in ChMKO mice following intraperitoneal injection of 1.0 U/kg body weight insulin, n = 9 biological replicates, *d* value for F/F vs. ChMKO: 0min (0.63), 15min (0.22), 30min (0.44), 60min (0.37), 120min (0.13).

(R) Plasma TG and TC levels in ChMKO mice, n = 10 biological replicates, 2 technical replicates per sample, *d* value for F/F vs. ChMKO: TG (0.52), TC (0.29).

Figure S3. RNA-seq Analysis of the GC and SOL Muscles in ChMKO Mice.

(A) PCA of gene expression.

(B) Cluster analysis of gene expression profiles in the GC and SOL of ChMKO mice.

(C, D) GSEA analysis of DEGs in the GC and SOL of ChMKO mice compared with F/F controls, showing enrichment plots for Hallmark pathways.

Figure S4. Effects of Hypoxic Exposure on Skeletal Muscle Phenotype and ChREBP Expression.

(A-D) Bodyweight, lean/bodyweight ratio, fat/bodyweight ratio and BMD in ChMKO mice compared with F/F controls after hypoxic exposure, F/F norm, n = 9; F/F hypo, n = 7; ChMKO norm, n = 8; ChMKO

hypo, n = 10 biological replicates. Data were analyzed using two-way ANOVA followed by Bonferroni's multiple comparisons test, *d* value for F/F vs. ChMKO: Bodyweight (0.07, 0.38), lean/body weight (0.18, 0.04), fat/body weight (0.18, 0.01), BMD (0.28, 0.14) in Norm and Hypo group, respectively.

(E-F) The mRNA levels of *ChREBP-α*, *ChREBP-β* and *Pklr* in the intestine (n = 10 biological replicates) and liver (n = 5 biological replicates) after hypoxic exposure, 2 technical replicates per sample. Data were analyzed using unpaired two-tailed Student's t-test, with Welch's correction applied when variances were unequal, *d* value for Norm vs. Hypo: intestine (4.59, 6.51, 4.20, power = 1), liver (0.59, 0.72, 0.51) for *ChREBP-α*, *ChREBP-β* and *Pklr*, respectively.

(G) Venn diagram comparing hypoxia-induced F/F_DEGs and ChMKO_DEGs (24 commonly upregulated and 29 commonly downregulated genes).

(H) GO enrichment analysis of the 24 commonly upregulated DEGs in F/F_DEGs and ChMKO_DEGs. The top 10 most significantly enriched pathways in biological processes and cellular component are shown.

(I-J) GO enrichment analysis of 88 upregulated and 339 downregulated genes among the unique ChMKO_DEGs, respectively. The top 10 most significantly enriched biological processes are displayed.

Figure S5. Analysis of Chronic High-Fructose Diet on Skeletal Muscle Phenotype and ChREBP Expression.

(A) The mRNA levels of *ChREBP-α*, *ChREBP-β* and *Pklr* in the small intestine after 2 weeks of high-fructose diet feeding, n = 10 biological replicates, 2 technical replicates per sample, unpaired two-tailed Student's t-test, *d* value for CHOW vs. HFrD: *ChREBP-α* (1.52, power = 0.97), *ChREBP-β* (1.28, power = 0.92) and *Pklr* (2.37, power = 1).

(B-D) The mRNA levels of *ChREBP*, slow-twitch and fast-twitch myofiber marker in the GC muscle after 2 weeks of HFrD feeding, n = 10 biological replicates, 2 technical replicates per sample, Data were analyzed using unpaired two-tailed Student's t-test, with Welch's correction applied when variances were unequal, *d* value for CHOW vs. HFrD: *ChREBP* (0.26),*Myh7* (0.32), *Myh7b* (0.26), *Tnni1* (0.44), *Tnnt1* (0.12), *Tnnc1* (0.41), *Myh4* (0.77), *Tnni2* (0.27), *Tnnc2* (1.24, power = 0.94), *Tnnt3* (1.20, power = 0.92) .

(E) Bodyweight, fat/body weight and lean/body weight in ChMKO mice compared with F/F controls after 3 months HFrD, n = 10 biological replicates, unpaired two-tailed Student's t-test, *d* value for CHOW vs. HFrD: bodyweight (3.93), fat/body weight (3.07) and lean/body weight (3.03), power = 1.

(F) PCA of gene expression in the GC muscle of mice fed a high-fructose diet for 3 months.

(G) Volcano plot depicting DEGs in the CHOW_F/F vs. HFrD_F/F. Horizontal dashed line denotes the significance cutoff of *P* value; vertical dashed lines mark the log_2FC threshold of ±1 for differentially expressed genes.

(H) Heatmap showing the expression of myofiber marker in the GC muscle of mice fed a high-fructose diet for 3 months.

(I) GO enrichment analysis of the 39 commonly downregulated DEGs in HFrD_DEGs and Hypo_DEGs. The top 10 most significantly enriched pathways in biological processes and the only 2 significant pathways in cellular component are shown.

Figure S6. ChREBP Enhances Myogenic Differentiation in C2C12 Cells.

(A) The mRNA levels of total *ChREBP* in wild-type C2C12 cells at days 0,4,8,12,16, and 20 of myogenic differentiation, n = 5 biological replicates (independent cell culture wells), 2 technical replicates per

sample. Data were analyzed by one-way ANOVA with Dunnett's post-hoc test. The annotated P-value represents the comparison of Day 4 versus Day 0, $d = 1.52$, power = 0.97.

(B) The protein levels of ChREBP in wild-type C2C12 cells at days 0,4,8,12,16, and 20 of myogenic differentiation.

(C) GO enrichment analysis of 107 downregulated DEGs in Ch-OE cells. The top 10 most significantly enriched pathways in biological processes are shown.

(D) The mRNA levels of *ChREBP, Myh2, Myh1, Myl1, Myl2, Myl3, Tnnc1, Tnni1, Tnnt1, Tnnc2, Tnni2,* and *Tnnt3* in control and Ch-OE C2C12 cells at days 4, 6, and 8 of myogenic differentiation, n = 4 biological replicates, 2 technical replicates per sample. Data were analyzed using unpaired two-tailed Student's t-test, with Welch's correction applied when variances were unequal. Labeled P-values denote CON vs. Ch-OE comparisons within the same time point.

(E) The mRNA levels of key regulatory molecules *Pax7* and *Myod1* in control and Ch-OE C2C12 cells at days 4, 6, and 8 of myogenic differentiation, n = 4 biological replicates, 2 technical replicates per sample.

(F) The protein levels of ChREBP, MyHC, DESMIN, MYOG, and MYOD1 in control and Ch-OE C2C12 cells at days 0, 4, 8, and 12 of myogenic differentiation.

Figure S7. Analysis of the Function of ChREBP in Primary Skeletal Muscle Satellite Cells.

(A) Heatmap showing the expression of key regulatory factors and ChREBP at different differentiation stages of MuSCs in the GSE59272 dataset.

(B) Following infection of the Cre adenovirus, Western blotting was performed to determine the protein expression level of ChREBP in myotubes at days 3 of differentiation after viral infection of MuSCs.

(C) Myosin positive area in Fig. 6G was quantified using ImageJ software. One microscopic field of view was captured per biological replicate, with four fields quantified in total per group, $d = 0.01$.

(D-G) The mRNA levels of *ChREBP, Pax7, Myod1* and *Myog* in MuSCs on day 3 of myogenic differentiation, n = 6 biological replicates, 2 technical replicates per sample, Welch's t -test, d values for Ad-GFP vs. Ad-CRE: *ChREBP* (2.91, power = 1), *Pax7* (0.45), *Myod1* (0.54), *Myog* (0.45).

Figure S8. Detection of Inflammatory Cytokines in a ChREBP-Overexpressing Skeletal Muscle Injury Model.

(A-C) IL-13, TNF-α and IFN-γ detection of TA muscle at 7 days post CTX injection, n = 5 biological replicates, 2 technical replicates per sample. Data were analyzed using two-way ANOVA followed by Bonferroni's multiple comparisons test, d values for AAV-GFP vs. AAV-ChREBP: IL-13 (0.39, 0.33), TNF-α (0.67, 0.40) and IFN-γ (0.39, 0.15) in CON and CTX group, respectively.
