# Supplementary figures and images for: ChREBP Is Dispensable for Myofiber Type Switch but Promotes Skeletal Muscle Regeneration
Source: Nutrients. 2026 Jun 21;18(12):2012. doi: 10.3390/nu18122012 (PMC13305853; doi:10.3390/nu18122012)

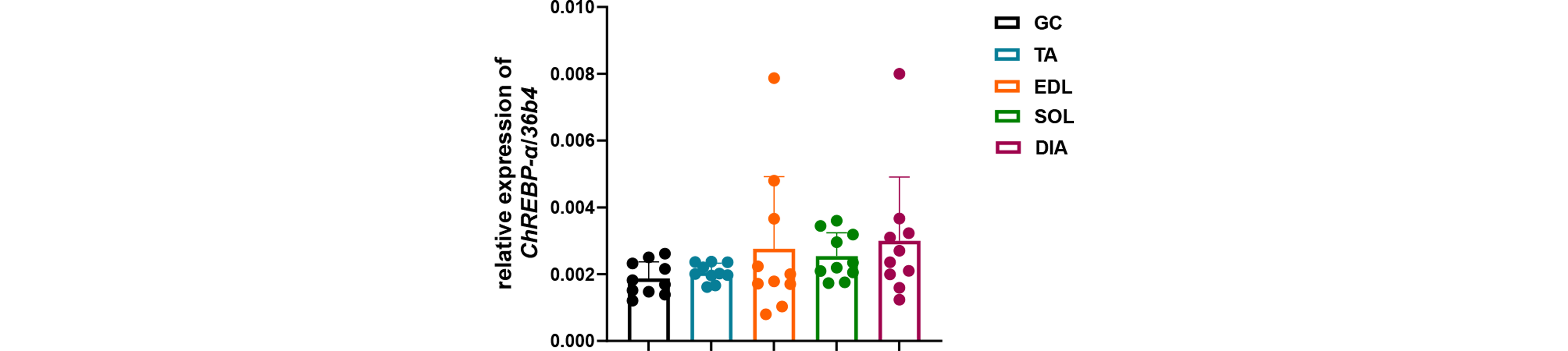

Supplement: Supplementary file 1 [file nutrients-18-02012-s001.zip › Fig-S1.png]

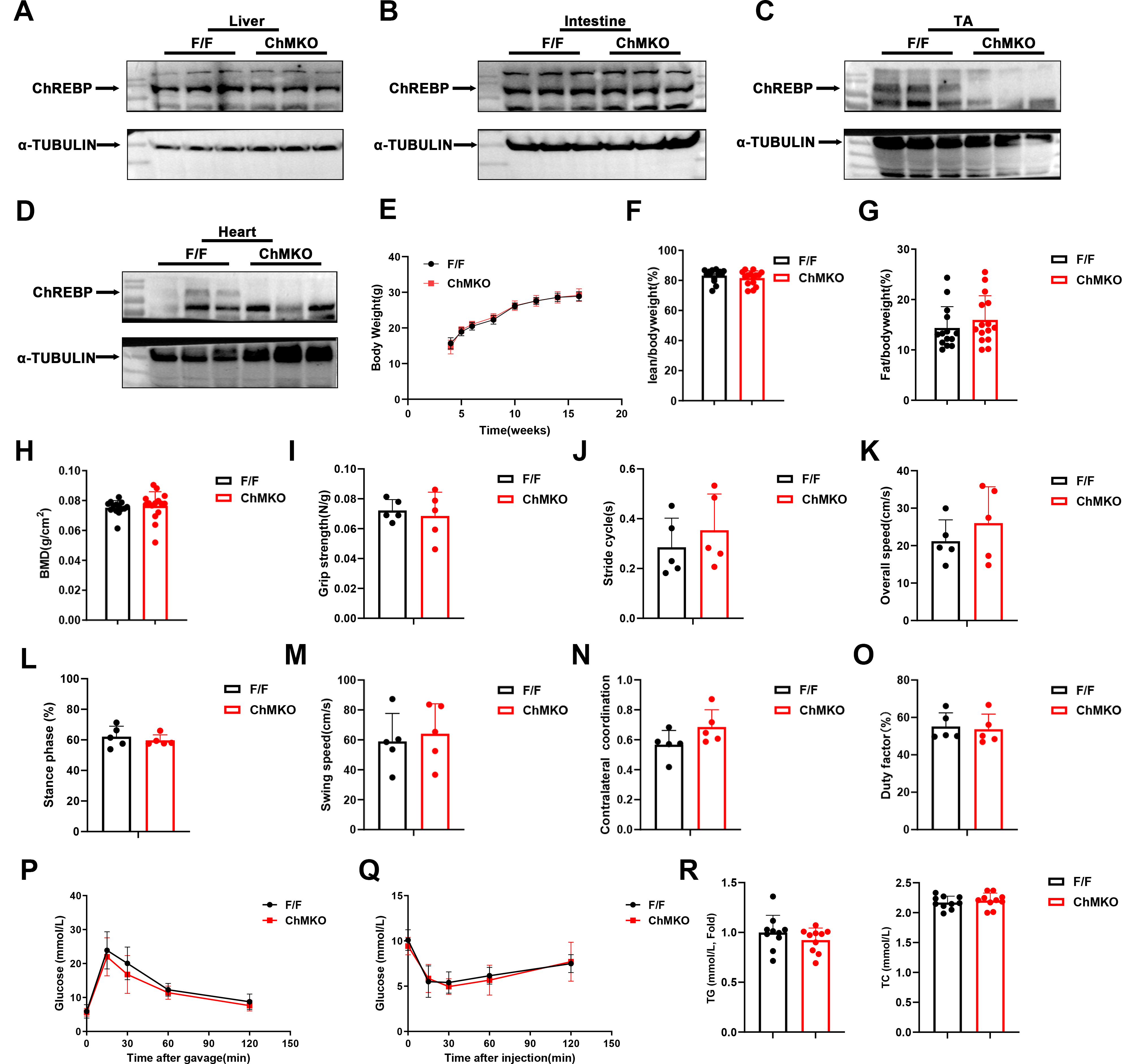

Supplement: Supplementary file 1 [file nutrients-18-02012-s001.zip › Fig-S2.png]

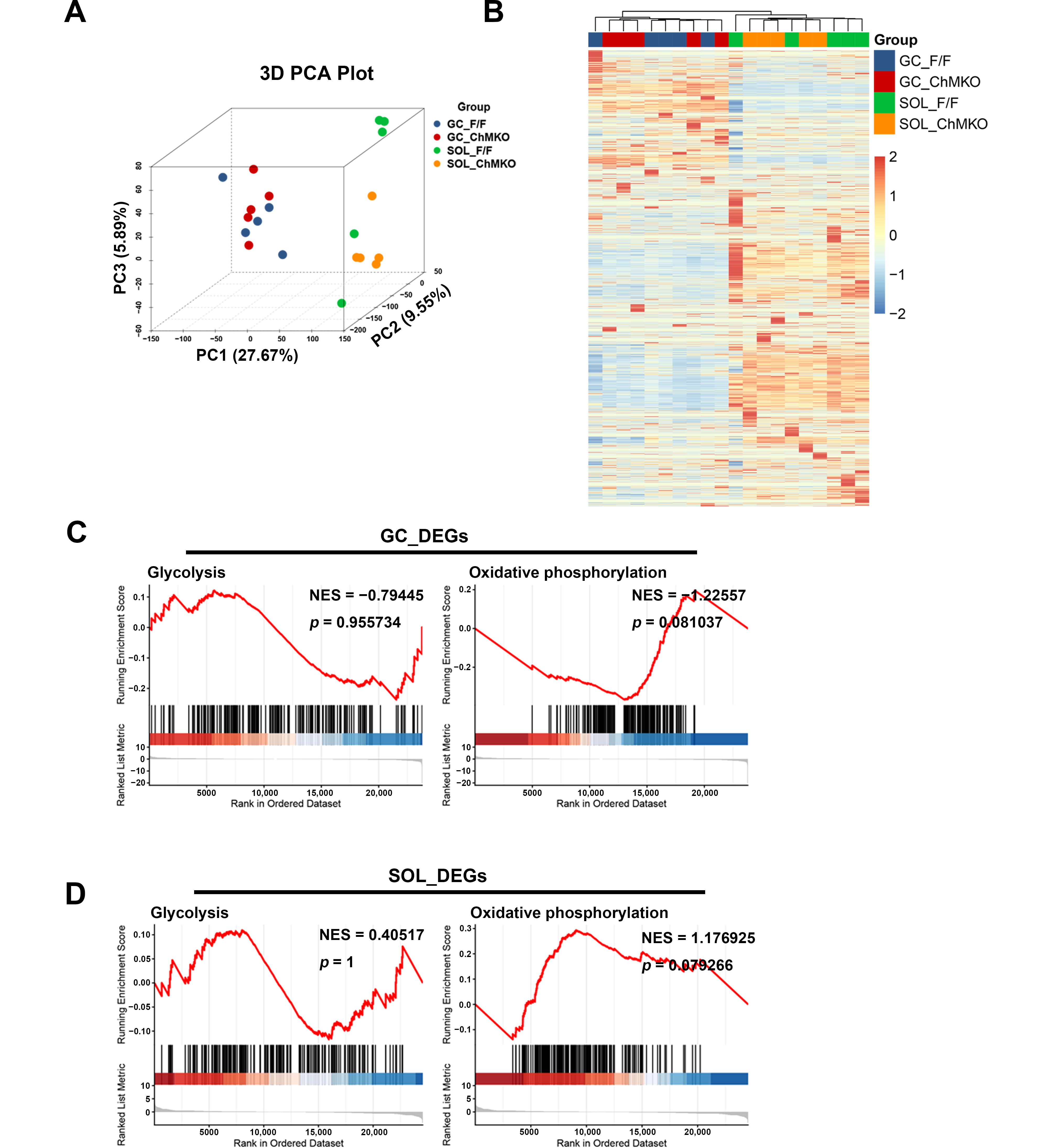

Supplement: Supplementary file 1 [file nutrients-18-02012-s001.zip › Fig-S3.png]

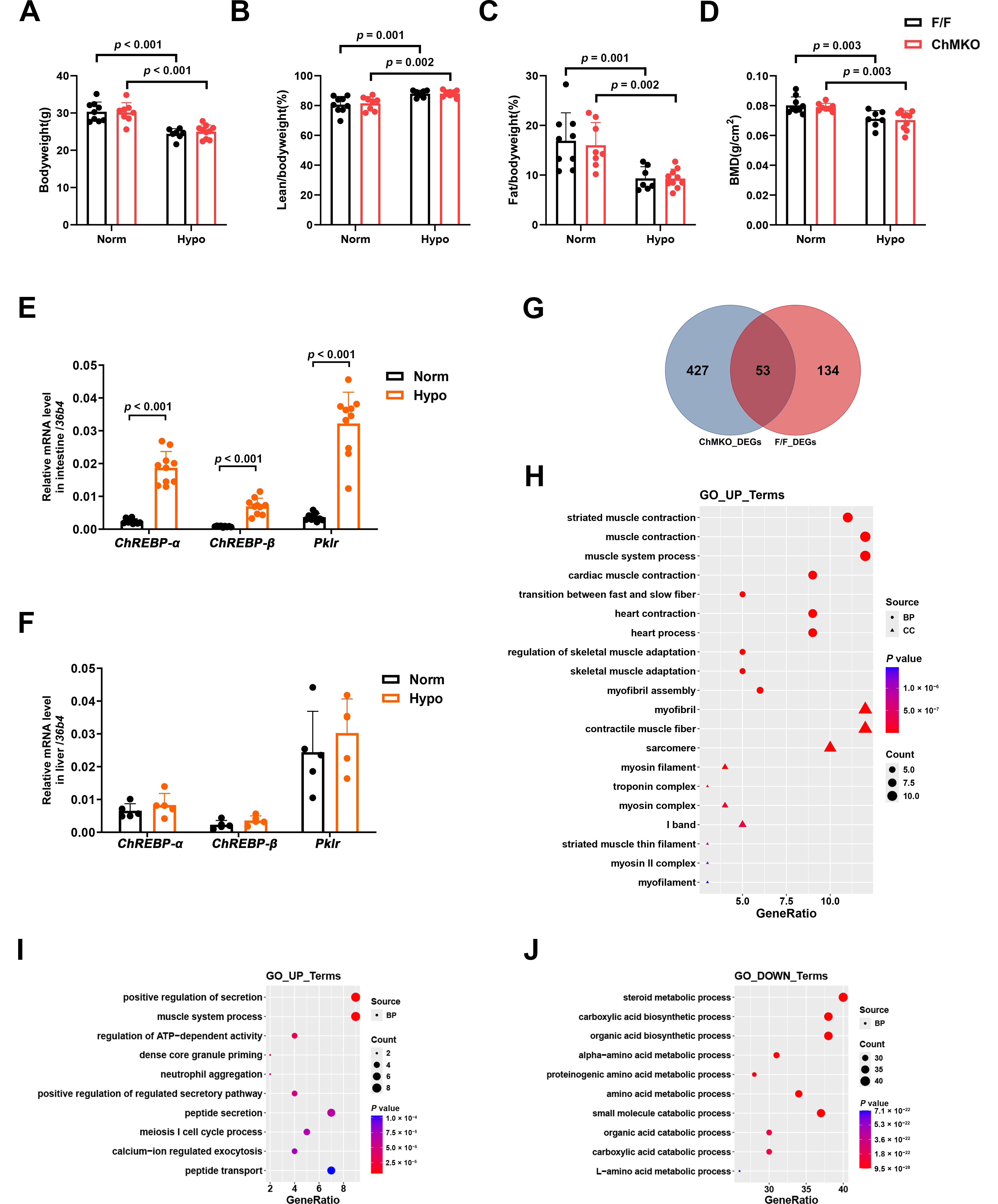

Supplement: Supplementary file 1 [file nutrients-18-02012-s001.zip › Fig-S4.png]

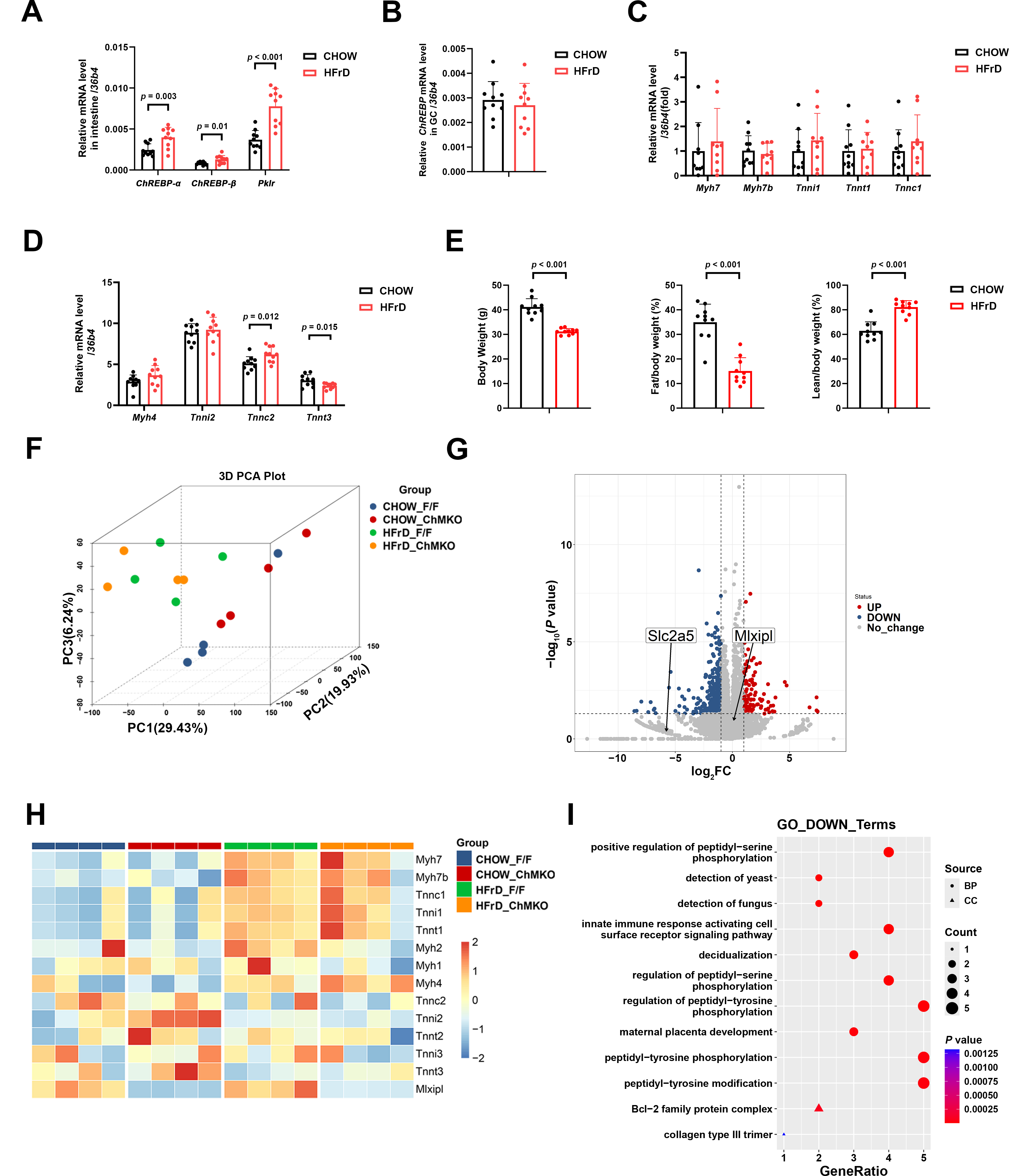

Supplement: Supplementary file 1 [file nutrients-18-02012-s001.zip › Fig-S5.png]

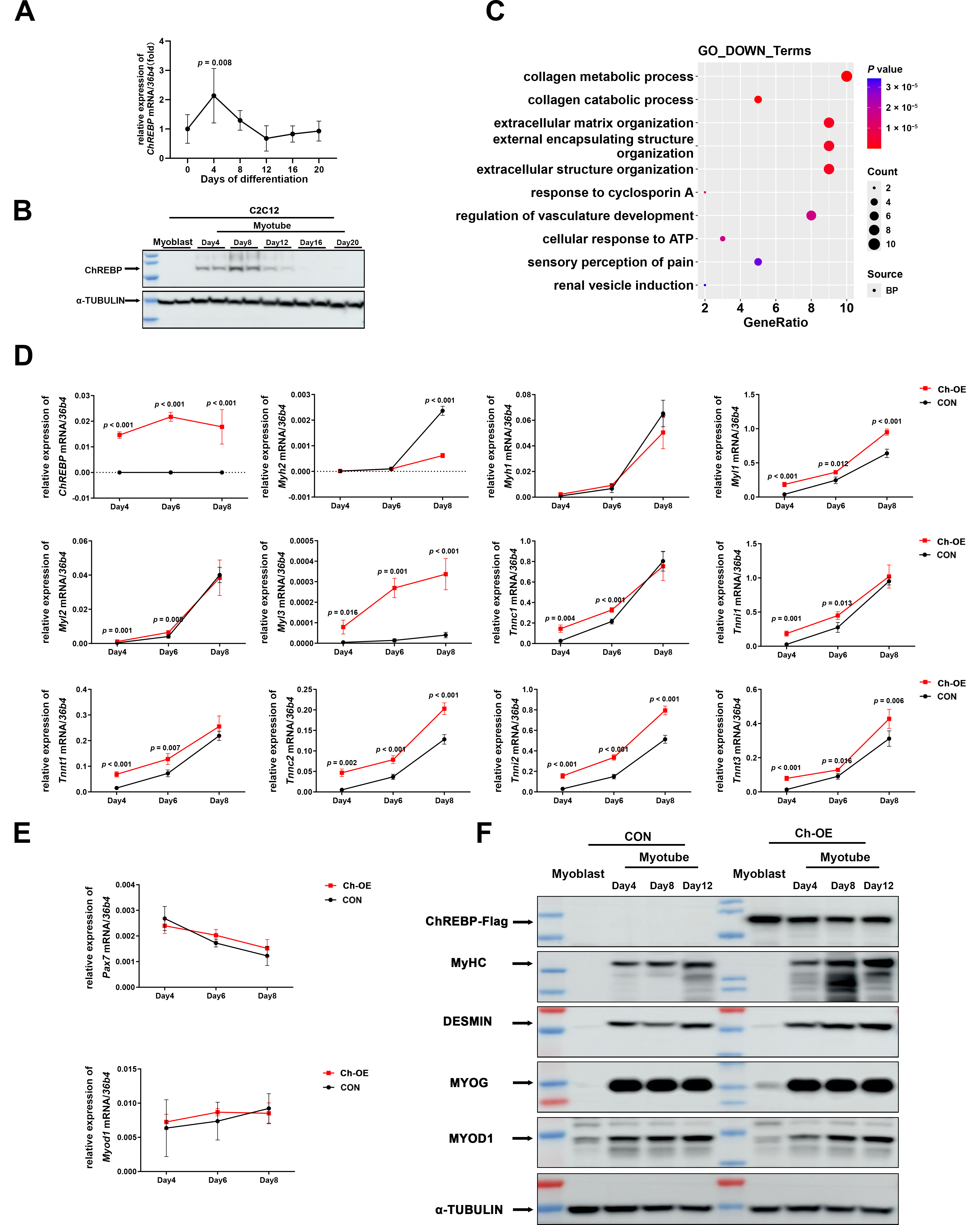

Supplement: Supplementary file 1 [file nutrients-18-02012-s001.zip › Fig-S6.png]

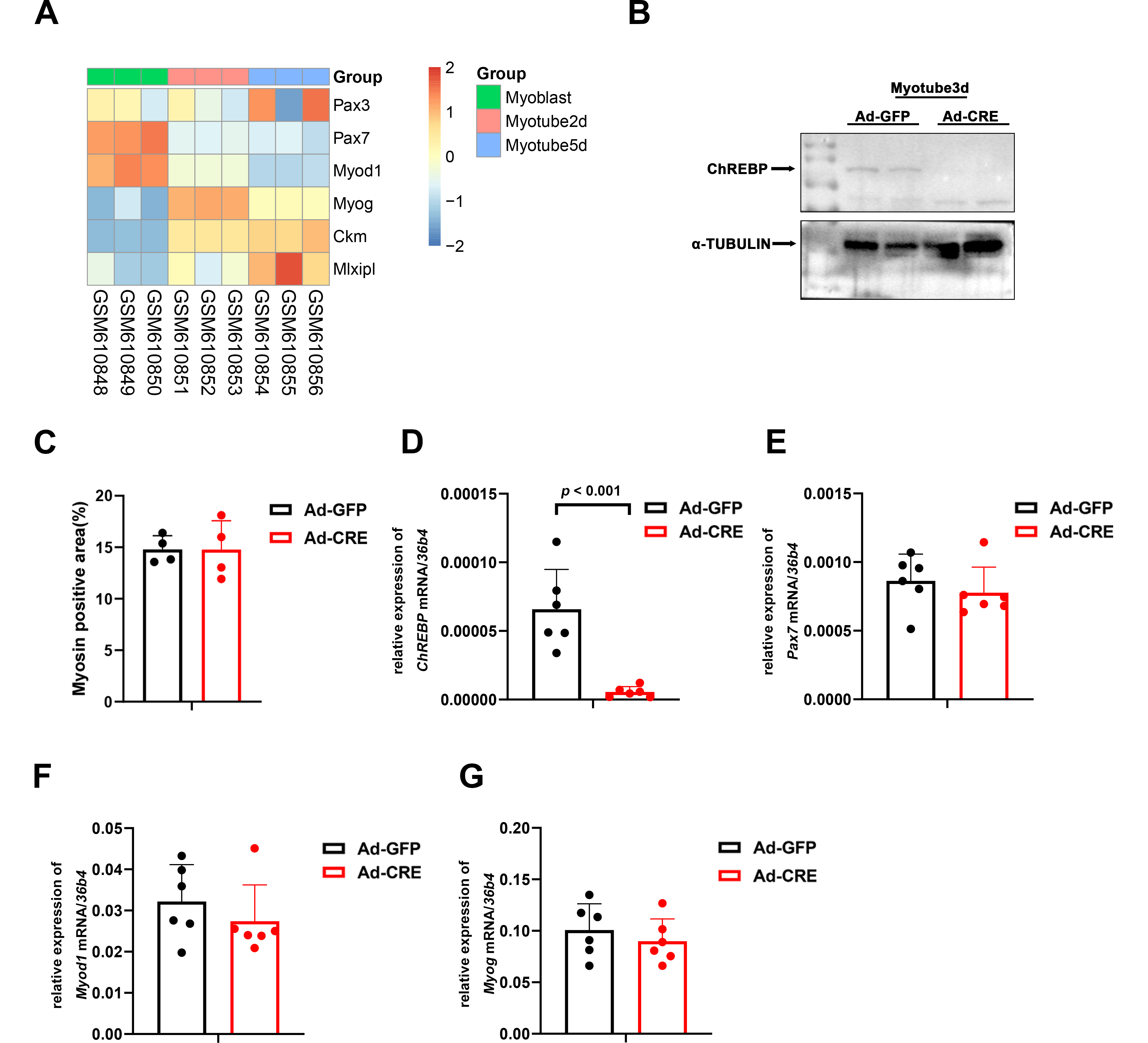

Supplement: Supplementary file 1 [file nutrients-18-02012-s001.zip › Fig-S7.png]

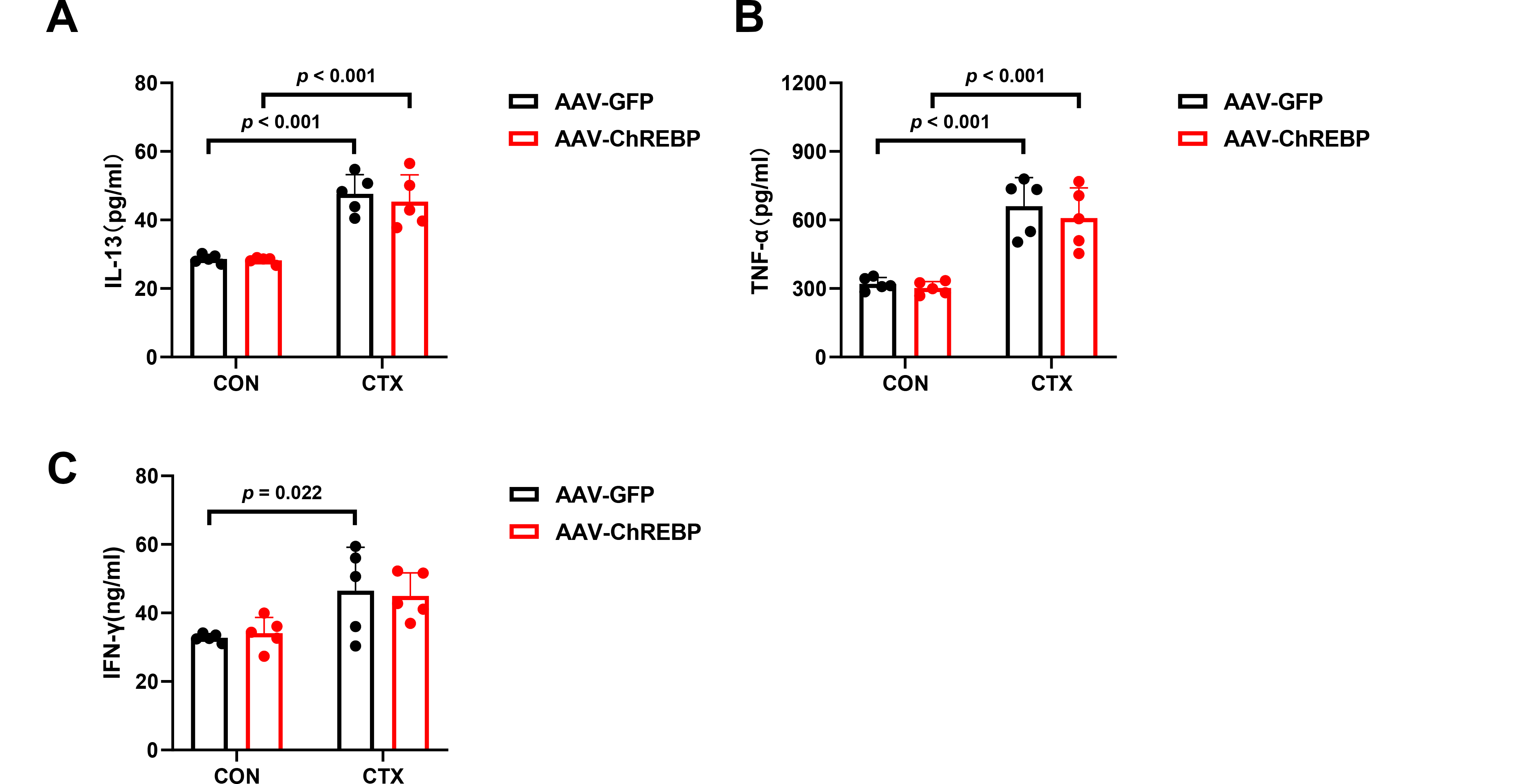

Supplement: Supplementary file 1 [file nutrients-18-02012-s001.zip › Fig-S8.png]
